# Mapping hand function with simultaneous brain–spinal cord functional MRI

**DOI:** 10.1162/IMAG.a.159

**Published:** 2025-10-03

**Authors:** Valeria Oliva, Sandrine Bédard, Merve Kaptan, Dario Pfyffer, Brett Chy, Susanna Aufrichtig, Nazrawit Berhe, Akshay S. Chaudhari, Suzanne Tharin, Serena S. Hu, John Ratliff, Zachary A. Smith, Andrew C. Smith, Gary H. Glover, Sean Mackey, Christine S.W. Law, Kenneth A. Weber II

**Affiliations:** Division of Pain Medicine, Department of Anesthesiology, Perioperative and Pain Medicine, Stanford University School of Medicine, Palo Alto, CA, United States; Center for Behavioral Sciences and Mental Health, Italian National Institute of Health, Rome, Italy; NeuroPoly Lab, Institute of Biomedical Engineering, Polytechnique Montréal, Montréal, Québec, Canada; Department of Radiology, Stanford University School of Medicine, Palo Alto, CA, United States; Department of Neurosurgery, Stanford University School of Medicine, Palo Alto, CA, United States; Department of Surgery, Veterans Affairs Palo Alto Health Care System, Palo Alto, CA, United States; Department of Orthopaedic Surgery, Stanford University School of Medicine, Palo Alto, CA, United States; Department of Neurosurgery, University of Oklahoma Health Sciences Center, Oklahoma City, OK, United States; Physical Therapy Program, Department of Physical Medicine and Rehabilitation, School of Medicine, University of Colorado, Aurora, CO, United States

**Keywords:** functional MRI, brain, spinal cord, musculoskeletal and neural physiological phenomena, motor activity, hand strength

## Abstract

Hand motor control depends on intricate brain–spinal cord interactions that regulate muscle activity. Hand function can be disrupted by injury to the brain, spinal cord, and peripheral nerves leading to weakness and impaired coordination. Functional MRI (fMRI) can map motor-related neural activity and potentially characterize the mechanisms underlying hand weakness and diminished coordination. Although brain motor control has been extensively studied, spinal cord mechanisms remain less explored. Here we use simultaneous brain–spinal cord fMRI to map neural activity related to hand strength and dexterity across the central nervous system using force matching and finger tapping tasks. We performed simultaneous brain–spinal cord fMRI in 28 right-handed healthy volunteers (age: 40.0 ± 13.8 years, 14 females, 14 males) using a 3T GE scanner. Participants performed a force-matching task at 10%, 20%, and 30% of maximum voluntary contraction. For the finger tapping task, participants completed button presses for three task levels: single-digit response with the second digit only, single-digit response with all digits in a sequential order, and single-digit response with all digits in a random order. Brain and spinal cord images were processed separately and assessed both activations and deactivations. Region of interest (ROI) analyses were also conducted to explore localized changes in activation across the task levels. Both tasks elicited activation in motor and sensory regions of the brain and spinal cord, with graded responses in the left primary motor (M1), left primary sensory (S1) cortex, and right spinal cord gray matter across task levels. Deactivation of the right M1 and S1 was also present for both tasks. Deactivation of the left spinal cord gray matter that scaled with task level was seen in the force matching task. The ROI analysis findings complemented the group level activity maps. Our study provides a detailed map of brain–spinal cord interactions in hand function, revealing graded neural activation and deactivation patterns across motor and sensory regions. Right M1 deactivation is likely evidence of interhemispheric inhibition that restricts extraneous motor output during unilateral tasks. For force matching, the deactivation of the left ventral and dorsal horns of the spinal cord provides the first evidence that the inhibition of motor areas during a unilateral motor task extends to the spinal cord. Whether this inhibition results from direct descending modulation from the brain or interneuronal inhibition in the spinal cord remains to be interrogated. These findings expand our understanding of central motor control mechanisms and could inform rehabilitation strategies for individuals with motor impairments. This approach may offer a foundation for studying motor dysfunction in conditions such as stroke, spinal cord injury, and neurodegenerative diseases.

## 1 Introduction

Hand function, ranging from gross grasping to precision movements, depends on coordinated interactions between the brain, spinal cord, and peripheral muscles. Cortical (e.g., primary motor (M1), primary sensory (S1), supplementary motor area (SMA), premotor cortices) and subcortical (e.g., basal ganglia, cerebellum) structures contribute to planning and executing voluntary movements. These brain regions transform a movement plan from real-world coordinates to muscle-specific motor commands which descend the spinal cord and recruit motor neurons leading to the appropriate muscle contractions (Castiello & Begliomini, 2008; Witt et al., 2008). Despite extensive research on the brain mechanisms of hand function, it is necessary to better understand how spinal cord activity integrates with brain motor control. Injuries or dysfunction at these levels can cause weakness and impaired coordination, yet distinguishing between cortical, subcortical, and spinal contributions remains a challenge. Simultaneous brain–spinal cord functional MRI (fMRI) offers a unique opportunity to resolve these gaps by mapping functional interactions across the neuraxis.

fMRI has revolutionized our understanding of brain motor control, revealing cortical reorganization in stroke and Parkinson’s disease and informing rehabilitation strategies (Binkofski et al., 1999; Filippi et al., 2019; Grefkes & Fink, 2011; Hou et al., 2016; Johansen-Berg et al., 2002; Leskinen et al., 2024). Recent advances—such as improved shimming, reduced field-of-view imaging, and specialized spinal cord imaging techniques—now enable fMRI to probe spinal cord function, bridging the gap between brain and spinal motor research (Finsterbusch et al., 2012; Fratini et al., 2014; Giove et al., 2004; Islam et al., 2019; Kaptan et al., 2024). The development of simultaneous brain–spinal cord fMRI now allows for the comprehensive mapping of motor function across the central nervous system (Vahdat et al., 2015). While prior studies have separately examined cortical and spinal motor activity, a unified view of how the brain and spinal cord interact during voluntary movement remains elusive (Giulietti et al., 2008; Hemmerling et al., 2023; Hernandez-Charpak et al., 2025; Kinany et al., 2023; Maieron et al., 2007; Stroman & Ryner, 2001; Weber et al., 2016).

Here we build on this work and use simultaneous brain–spinal cord fMRI during force matching (i.e., hand strength) and finger tapping (i.e., dexterity) tasks, each at three task levels, to map graded motor and sensory activity across the central nervous system. Force matching and finger tapping tasks have been used for decades to assess motor function in healthy individuals (Barut et al., 2013; Maieron et al., 2007; Nalçaci et al., 2001) and in patients with impaired hand function (Heller et al., 1987; Jobbágy et al., 2005; Notermans et al., 1994). We expected to see graded activity across the task levels specific to the motor, temporal, and attentional demands of the motor tasks. A deeper understanding of central motor control mechanisms can improve strategies to maintain hand function across the lifespan, our understanding of disease mechanisms, the tracking of disease progression and recovery, and the development of improved diagnostic strategies and novel therapies.

## 2 Methods

### 2.1 Participants

Twenty-eight right-handed healthy volunteers (age = 40.0 ± 13.8 years, 14 females, 14 males) were recruited between September 2023 and March 2024. The number of participants enrolled was determined from previous simultaneous brain–spinal cord fMRI motor studies (Khatibi et al., 2022; Vahdat et al., 2015). No *a priori* power analysis was performed. Exclusion criteria included any contraindications to MRI, pain conditions, previous spine surgery, and major medical, psychiatric, and neurological conditions. Eligible volunteers received an overview of the study protocol, and then gave written informed consent. Participants were compensated for participating in the study. The study was approved by Stanford University’s Institutional Review Board.

### 2.2 Hand function

We assessed handedness using the Edinburgh Handedness Inventory Short Form (Veale, 2014). We measured right hand grip strength (Jamar Plus+ Digital Dynamometer, Performance Health Supply, Inc., Cedarburg, WI, USA) and dexterity (Jamar 9 Hole Peg Test Kit, Performance Health Supply, Inc., Cedarburg, WI, USA) using the normative scores from the NIH Toolbox motor battery, which account for age, sex, education level, race, and ethnicity (Reuben et al., 2013). Upper extremity physical function was also assessed using the Quick DASH outcome measure (Beaton et al., 2005) and PROMIS Upper Extremity Short Form 7a (version = 2.1) (Kaat et al., 2019).

### 2.3 Functional MRI experiments

For the force matching task, participants gripped an MRI-compatible dynamometer (BIOPAC Systems, Inc., Goleta, CA, USA) with their right hand at 10%, 20%, and 30% of their maximum voluntary contraction corresponding to low, medium, and high task levels, respectively. Visual cues were provided with Eprime (Version 2.0, Psychology Software Tools, Pittsburgh, PA) using a vertical white bar and a white square bracket that spanned the target force ±1 kgf. The white bar turned green when the force applied to the dynamometer was within the square bracket. The force measures were sent to Eprime in real time using BIOPAC’s network data transfer and a socket connection (BIOPAC, AcqKnowledge Data Acquisition and Analysis Software, Goleta, CA, USA). Immediately prior to starting the force matching experiment, maximum voluntary contraction was measured by asking the participant on the scanner bed to squeeze the dynamometer as hard as possible for a count of three with verbal encouragement.

For the finger tapping task, participants responded by pressing buttons on an MRI-compatible five button fiber optic response pad (Pyka 5 Button Handheld, Current Designs, Inc., PA, USA) with their right hand. Visual cues were provided with Eprime by placing a white circle at the distal aspect of the digits of a white hand, indicating when and which button to press. The circles appeared at a rate of 1 Hz, remained visible for 900 ms, and turned green if the correct button was pressed. The experiment had three task levels: a single-digit response with the second digit only, a single-digit response with all digits in a sequential order, and a single-digit response with all digits in a random order, corresponding to low, medium, and high task levels, respectively.

**Fig. 1.**
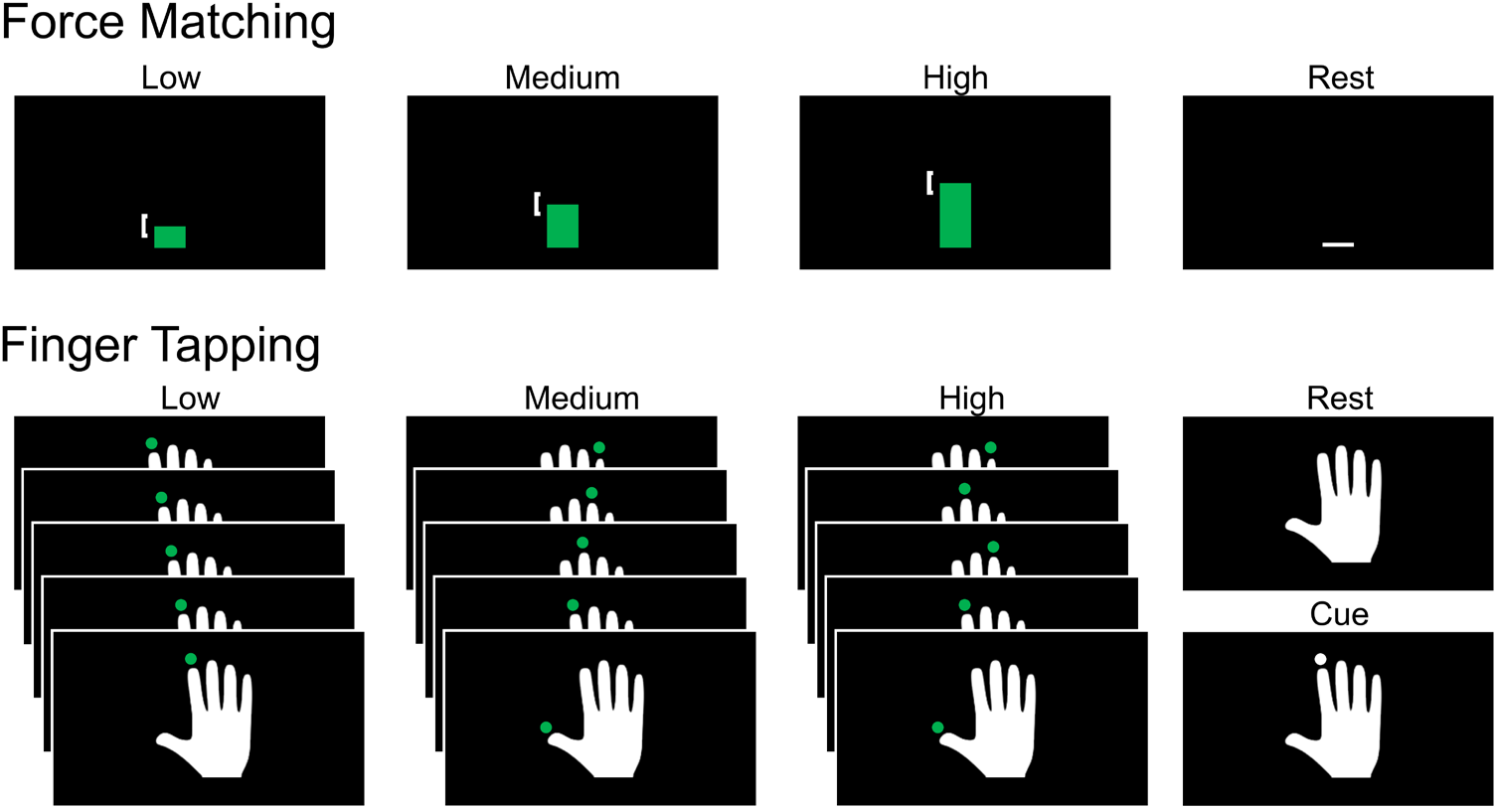
Visual cues for the force matching and finger tapping tasks. For the force matching task, participants continuously gripped an MRI-compatible dynamometer with their right hand to match 10%, 20%, and 30% of their maximum voluntary contraction, corresponding to low, medium, and high task levels, respectively. Visual cues were provided with a vertical white bar and a white square bracket that spanned the target force ±1 kgf. The white bar turned green when the force applied to the dynamometer was within the square bracket. For the finger tapping task, participants responded by pressing buttons on an MRI-compatible five button response pad with their right hand. Visual cues were provided by placing a white circle at the distal aspect of the digits of a white hand, indicating when and which button to press. The circles appeared at a rate of 1 Hz, remained visible for 900 ms, and turned green if the correct button was pressed. The experiment had three task levels: a single-digit response with the second digit only, a single-digit response with all digits in a sequential order, and a single-digit response with all digits in a random order, corresponding to low, medium, and high task levels, respectively.

The force matching and finger tapping tasks started with an initial 15 s rest period. Then, ten 15 s trials were performed at each task level with a 15 s rest period after each trial. A total of 30 trials were performed in each experiment. The order of the experiments and the task levels within each experiment were pseudorandomized. Figure 1 summarizes the visual cues for both force matching and finger tapping tasks.

### 2.4 Brain and spinal cord image acquisition

Brain and spinal cord imaging was performed on a 3T GE SIGNA Premier scanner equipped with a 21-channel neurovascular coil. Participants were briefly trained on the force matching and finger tapping tasks in the scanner room before being placed supine on the scanner bed. Participants were positioned to ensure maximum quality of the spinal cord functional images (Cohen-Adad et al., 2021). The cervical spine was positioned as straight as possible using a cushion below their head to minimize the cervical lordosis. This ensures that axial slices are orthogonal to the spinal cord centerline, thus minimizing partial volume effect (Cohen-Adad et al., 2021). A SatPad^TM^ cervical collar was used to increase the magnetic field homogeneity across the cervical spine and further reduce motion during scanning (Maehara et al., 2014). Physiological data were collected with a pulse oximeter attached to the left index finger and a respiratory belt around the abdomen. Functional imaging was performed using a simultaneous T2*-weighted brain–spinal cord sequence developed by our team (Islam et al., 2019) in separate 15-min runs for the finger tapping and force matching tasks. Briefly, the sequence uses separate field-of-views (FOVs) for the brain (spatial-spectral pulse, 30 slices, full field of view, 3.43 mm × 3.43 mm × 5.00 mm) and cervical spinal cord (2D spatially selective reduced FOV pulse, 15 slices, 1.25 mm × 1.25 mm × 5.00 mm). We aligned the brain FOV axially, while the spinal cord FOV was centered at the C5-C6 intervertebral disk and oriented obliquely such that the slices were perpendicular to the spinal cord. Due to decreasing signal in the inferior cervical spine caused by increasing distance from the receiver coil and proximity to the lungs with associated B_0_ inhomogeneity, the investigation of spinal cord activity was limited to the C5 to C7 spinal cord segments. Axial (FOV = 220 mm × 220 mm, matrix size = 128 × 128, ΔTE = 0.5 ms) and sagittal (FOV = 300 mm × 300 mm, matrix size = 256 × 64, ΔTE = 0.5 ms) field maps were collected to calculate the *x* and *y* shim values, while the optimal *z* shim was manually selected by the imaging technologists. Dynamic x, y, and z shimming was then applied to each brain and spinal cord slice to maximize the signal intensity and minimize distortions. Functional images were acquired with 2.6 s TR (TE = 30 ms, flip angle = 80, bandwidth = 250 kHz, GRAPPA acceleration = 2, phase encoding direction = A/P). A two-volume acquisition with opposite phase-encoding direction was collected immediately following the functional runs for distortion correction of the brain functional images. Finally, a T1-weighted brain image (3D FSPGR sequence, 1.0 mm × 1.0 mm × 1.0 mm) and a T2-weighted spinal cord image were collected (3D turbo spin-echo, 0.8 mm × 0.8 mm × 0.8 mm) for registration and spatial normalization (Cohen-Adad et al., 2021).

### 2.5 Brain and spinal cord image processing

Brain and spinal cord functional images for both the force matching and finger tapping tasks were preprocessed and analyzed with separate but analogous pipelines. Preprocessing was performed using commands from Advanced Normalization Tools (ANTS) (version = 2.5.1) (Avants et al., 2011), the Oxford Center for fMRI of the Brain’s (FMRIB) Software Library (FSL) (version = 6.0) (Jenkinson et al., 2012), and the Spinal Cord Toolbox (SCT) (version = 6.2) (De Leener et al., 2017). All preprocessing and analysis code is available on GitHub (https://github.com/NeuromuscularInsightLab/Oliva_Mapping_Hand_Functon_Imaging_Neuroscience_2025).

#### 2.5.1 Brain preprocessing

The T1-weighted structural image was brain extracted using antsBrainExtraction (Avants et al., 2011), and then fast was used to segment the brain-extracted image into gray matter (GM), white matter (WM), and cerebrospinal fluid (CSF) (Zhang et al., 2001). The functional images were motion corrected with mcflirt before correction for susceptibility induced distortions using topup. The WM and CSF masks, generated from the segmentation of the T1-weighted brain image, were initially registered to the functional images using flirt (Jenkinson & Smith, 2001), and the first principal components from each were extracted to create volume-wise WM and CSF nuisance regressors with fslmeants. Slice-wise cardiac and respiratory noise regressors were also generated using PNM (Brooks et al., 2008) for a total of 32 regressors. These regressors were then included in a general linear model using feat along with the six motion parameters to remove WM, CSF, physiological, and motion-related noise from the brain functional time series. High-pass temporal filtering (100 s cut-off) was also applied at this stage to the functional series and the noise regressors.

The mean brain functional image was co-registered to the T1-weighted structural images using flirt with six degrees of freedom, and then the brain-extracted T1-weighted structural image was normalized to MNI152 template space (2 mm × 2 mm × 2 mm) using non-linear registration with 12 degrees of freedom and a 10 mm warp field in fnirt (Andersson et al., 2007). These transformations were concatenated, and the functional times series was then warped to standard space using applywarp. The functional images were spatially smoothed with a 5 mm × 5 mm × 5 mm full width half maximum (FWHM) with SUSAN. These preprocessing steps generated denoised functional brain images in a standard space to be used as inputs for the first level analyses.

#### 2.5.2 Spinal cord preprocessing

Motion correction of the functional spinal cord time series was performed using flirt. A mask around the spinal canal was generated by combining and dilating the automatic segmentations of the CSF and spinal cord output by sct_propseg and used to exclude areas outside of the spinal column (De Leener et al., 2014). The volumes were then aligned with the mid-volume of the functional time series using two-dimensional rigid realignment of each axial slice, the mean image was then calculated, and the motion correction realignment was repeated aligning each volume to the mean image (Weber et al., 2014). At each step of motion correction, the functional time series was visually inspected.

The spinal cord was then segmented from the mean functional image of the motion-corrected time series with sct_deepseg (Bédard et al., 2025). A spinal canal mask was generated by combining the spinal cord and CSF segmentations output from sct_propseg, and a CSF mask was generated by subtracting the sct_deepseg spinal cord segmentation from the spinal canal segmentation. All segmentations were visually assessed using sct_qc and manually corrected as necessary.

The spinal cord from the T2-weighted structural image was segmented automatically using SCT’s contrast-agnostic segmentation method, sct_deepseg (Bédard et al., 2025), and the vertebral levels were automatically labeled using sct_label_vertebrae (Ullmann et al., 2014). The spinal cord T2-weighted image was then registered to the PAM50 spinal cord template using sct_register_to_template (De Leener et al., 2018). The PAM50 template was registered to the mean spinal cord functional image with sct_register_multimodal using the spinal cord segmentations, non-linear slice-wise transforms, and the T2-weighted structural image to PAM50 template transformation to initialize the registration. These transformations were concatenated, the PAM50 template was warped to the functional images using sct_warp_template, and a WM mask was generated by thresholding and binarizing the PAM50 WM probability atlas (threshold = 0.9).

Slice-wise WM and CSF nuisance noise regressors were generated by extracting the first principal components of the WM and CSF time series, respectively, with fslmeants. Slice-wise cardiac and respiratory noise regressors were generated using PNM (32 regressors) as well as slice-wise motion regressors (x translation, y translation, and z rotation). These regressors were included in a general linear model using feat to remove physiological- and motion-related noise from the spinal cord functional time series. High-pass temporal filtering (100 s cut-off) was also applied at this stage to the functional series and the noise regressors. The denoised functional time series was warped to PAM50 template space (0.5 mm × 0.5 mm × 0.5 mm). In the template space, smoothing was applied with a 2 mm × 2 mm × 5 mm FWHM Gaussian kernel, to preferentially smooth along the superior-inferior axis of the spinal cord. These preprocessing steps generated denoised functional spinal cord images in a standard space to be used as inputs for the subject level analyses. The temporal signal-to-noise ratio (tSNR) was similar for both tasks (Supplementary Fig. S1).

#### 2.5.3 Subject level analysis

Subject level analyses were performed identically for brain and spinal cord data using FEAT and included correction for temporal autocorrelations using FILM prewhitening. The force matching and finger tapping tasks were modeled using trial-wise boxcar regressors convolved with a double gamma hemodynamic response function. The trial-wise task activation maps from the preprocessed functional time series were then entered into a second level fixed effects model to create average subject level activity maps for each task level. A linear contrast across the task levels was also applied to map where the activity scaled linearly with task level (coefficients of -1, 0, and +1 for positive linear contrast and +1, 0, and -1 for negative linear contrast).

#### 2.5.4 Group level analysis

The second level activity brain maps from all contrasts were then entered into a third level mixed effects analysis (FLAME Stage 1 (Woolrich et al., 2004)) to generate group activity maps with age and sex included as covariates of no interest. The group level brain activity was defined using a voxel-wise threshold of Z score > 3.10 with a cluster significance threshold of p < 0.05 to correct for multiple comparisons. *A* mixed effects analysis was planned *a priori* for the group level spinal cord analysis. However, significant group level spinal cord activity was not consistently present with a mixed effects analysis, and instead, the reported group level spinal cord activity maps were generated from a fixed effects analysis. Group level spinal cord activity was defined using a voxel-wise threshold of Z score > 2.30 with a cluster significance threshold of p < 0.05 to correct for multiple comparisons. The number of active voxels and the average Z score of the active voxels were extracted from each task level at the group level to assess the spatial extent and magnitude of the activity. Both activations (i.e., positive signal change) and deactivations (i.e., negative signal change) were assessed. For the spinal cord, analyses were confined to the region of intersection of the subject level maps, which spanned the C5 to C7 spinal cord segment levels.

### 2.6 Spinal cord spatial analysis

To summarize the localization of the activity, left-right (LR) indices at the group level were calculated by dividing the difference in the number of active voxels between the respective hemicords by the sum (number of active voxels in the entire spinal cord) (Seghier, 2008). A value of +1.00 indicates that all active voxels were in the left hemicord while a value of –1.00 indicates that all active voxels were in the right hemicord. The localization of the activity to the GM and WM was also assessed. As the volume of the WM is more than three times the volume of the GM, the ratio of the percentage of GM activation to the percentage of WM, GM-WM (GW) ratio, was calculated to account for the differences in volume. LR indices and GW ratios were assessed at the group level for each task level as well as the linear contrast.

### 2.7 Region of interest analysis

To explore local changes in the magnitude of activity across the three task levels, we performed a brain and spinal cord region of interest (ROI) analysis. For the brain ROIs, spheres (radius = 5 mm) were placed at the SMA, dorsal premotor cortex (dPMC), ventral premotor cortex (vPMC), primary motor cortex (M1), and primary somatosensory cortex (S1), regions responsible for human motor movement (Tanji, 2001). The selection of coordinates for the brain ROIs was initially guided by the meta-analysis of Mayka et al. (Mayka et al., 2006) and refined using the Mindboggle-101 DKT31 cortical atlas (Klein & Tourville, 2012) and Neurosynth (Yarkoni et al., 2011) association test maps (FDR < 0.01 thresholded) for the following terms: hand movements, supplementary motor, premotor, primary somatosensory. The brain coordinates in MNI space are provided in the Supplementary Material (Table S1). The spinal cord ROIs included the left and right gray matter from the PAM50 template (threshold > 0.50) spanning the C5 to C7 spinal cord segment levels. The average Z score within each ROI was extracted for each task level. Two-tailed one-sample t-tests were performed for each task level to identify positive (i.e., activation) or negative (i.e., deactivation) signal change within each ROI. Two-tailed paired t-tests were performed between each task level to identify differences in signal change between task levels for each ROI. Repeated measures, mixed effects linear models were performed to explore associations between left M1 activity and spinal cord GM activity across the task levels. Statistical significance for these exploratory analyses was set at an α < 0.05 without correction for multiple comparisons. Statistical analyses were performed in R via rpy2 (*rpy2: Python-R Bridge*).

### 2.8 Trial-wise analysis

For each subject and task level, the task error, the number of active voxels, and average Z score of the active voxels were extracted for each trial. Subject level activity for the brain and spinal cord was defined using a voxel-wise threshold of Z score >1.64 without cluster correction. For the force matching task, task error was assessed using the mean absolute percent error, which was calculated for each subject and trial for the low, medium, and high task levels. For the finger tapping task, task error was calculated as the percentage of missed taps in each trial for the low, medium, and high task levels. Repeated measures, mixed effects linear models were performed to explore linear changes in activity and task errors across the runs to identify the presence of fatigue or motor learning across the experiments. Each task level was investigated separately. Statistical significance for these exploratory analyses was set at an α < 0.05 without correction for multiple comparisons.

## 3 Results

### 3.1 Hand function

All participants were right handed based on the Edinburgh handedness short form (range = + 33 – +100 kgf). Average right hand grip strength was 22.1 ± 11.2 kgf (range = 6.3–48.8 kgf), and average NIH Toolbox dominant hand grip strength t-score was 45.2 ± 7.9 (range = 28–58). The average NIH Toolbox dominant hand dexterity t-score was 58.6 ± 10.5 (range = 37–77). The average Quick DASH outcome score was 3.2 ± 5.0 (range = 0–22.7), and the average PROMIS Upper Extremity score was 53.9 ± 5.3 (range = 40.6–57.1).

### 3.2 Force matching task

**Fig. 2.**
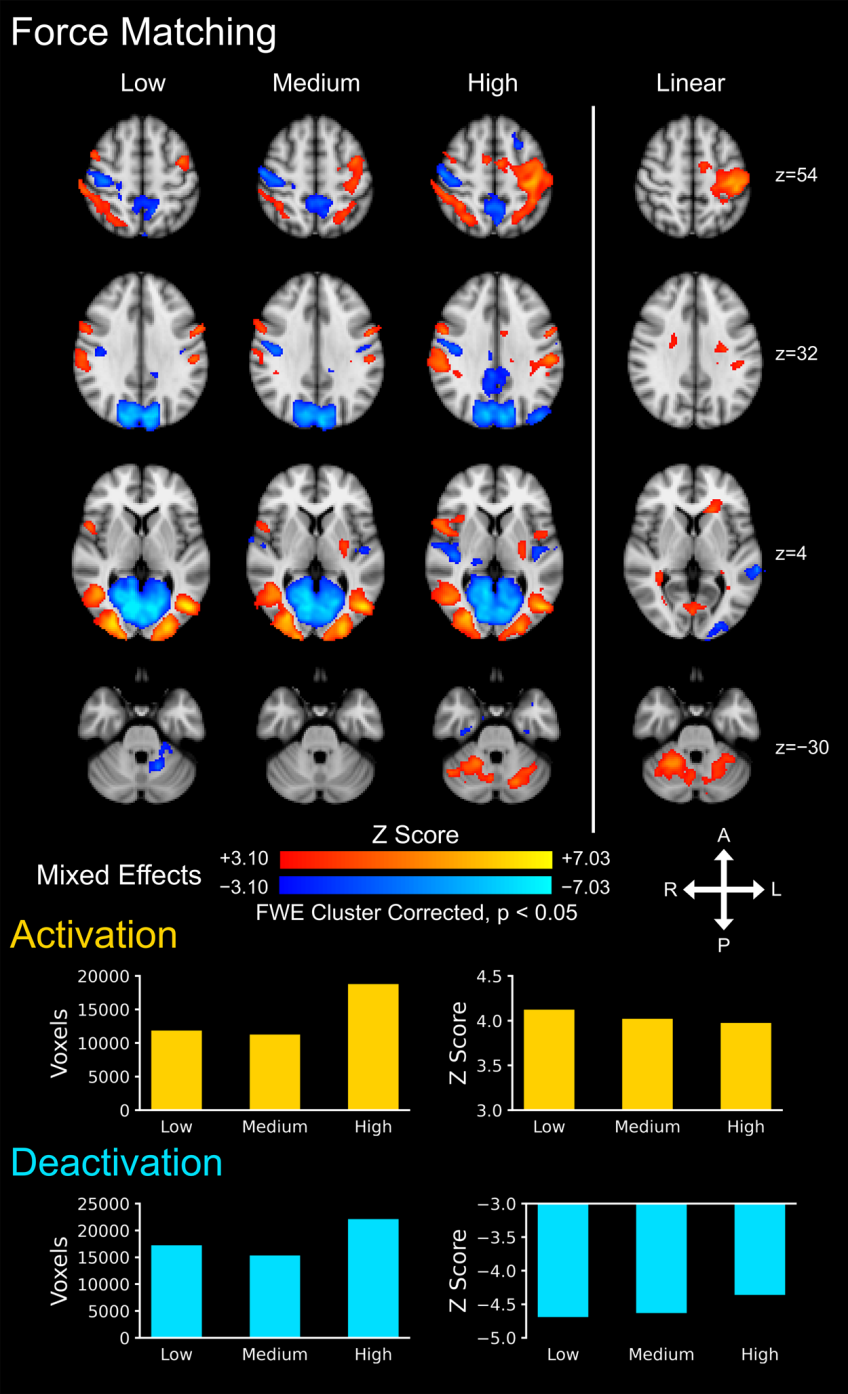
Group level brain activity for the force matching task across the three task levels: low, medium, and high. Activations (i.e., positive signal change) are shown in red–yellow and deactivations are shown in blue–light blue (i.e., negative signal change). A linear contrast across the task levels was applied to map where the signal linearly increases and decreases across the task levels. The number of active voxels and the average Z score of the active voxels are shown to summarize the spatial extent and magnitude of the activity across the three task levels. The activation maps were generated from a mixed effects analysis at the group level and were voxel-wise thresholded at a Z score >3.10 with a family-wise error (FWE) cluster correction threshold of p < 0.05. The background image is the MNI152 T1-weighted brain template. A = anterior, P = posterior, L = left, R = right.

Brain activity maps showed activations and deactivations in cortical and subcortical motor and sensory areas at each task level, including the contralateral M1, contralateral S1, and bilateral vPMC (mixed effects, Z > 3.10, cluster correction threshold p < 0.05) for the force matching task (Fig. 2). At the group level, the number of active voxels for the activations and deactivations was highest in the high task level. The average Z score of the activations and deactivations, in contrast, decreased with increasing task level.

**Fig. 3.**
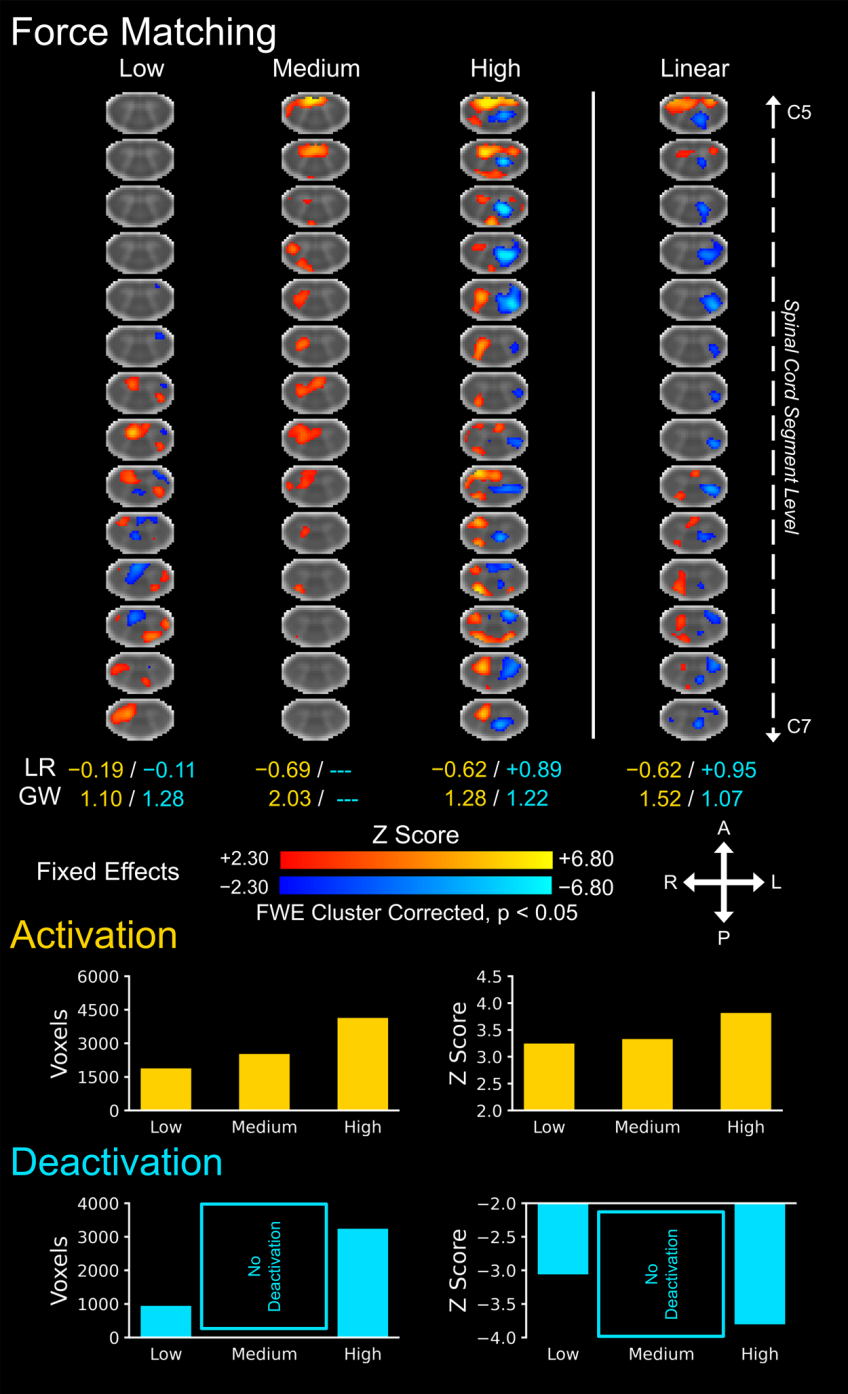
Group level spinal cord activity for the force matching task across the three task levels: low, medium, and high. Activations (i.e., positive signal change) are shown in red–yellow and deactivations are shown in blue–light blue (negative signal change). A linear contrast across the task levels was applied to map where the signal linearly increases and decreases across the task levels. The location of the activations and deactivations was assessed using the left–right (LR) index and gray matter–white matter (GW) ratio (--- = no activity, unable to calculate). The number of active voxels and the average Z score of the active voxels are shown to summarize the spatial extent and magnitude of the activity across the three task levels. The activation maps were generated from a fixed effects analysis at the group level and were voxel-wise thresholded at a Z score >2.30 with a family-wise error (FWE) cluster correction threshold of p < 0.05. The background image is the PAM50 T2*-weighted spinal cord template. Every fifth axial slice from the intersection of the subject level functional images is shown. A = anterior, P = posterior, L = left, R = right.

Spinal cord activity maps for the force matching task showed activation in the right ventral and dorsal horns for each task level (i.e., negative LR indices), and deactivation in the left ventral and dorsal horns at the high task level (Fig. 3). Activation in the right ventral and dorsal horns linearly increased with increasing task level while deactivation within the left ventral and dorsal horns linearly increased with increasing task level (fixed effects, Z > 2.30, cluster correction threshold p < 0.05). The activations and deactivations were localized more to the gray matter (i.e., GW ratio > 1). At the group level, the number of active voxels and the average Z score of the active voxels for activation increased across the task levels. See the Supplementary Material for spinal cord activity maps using a mixed effects analysis as well as maps with and without cluster correction (Supplementary Figs. S4, S6, and S8).

**Fig. 4.**
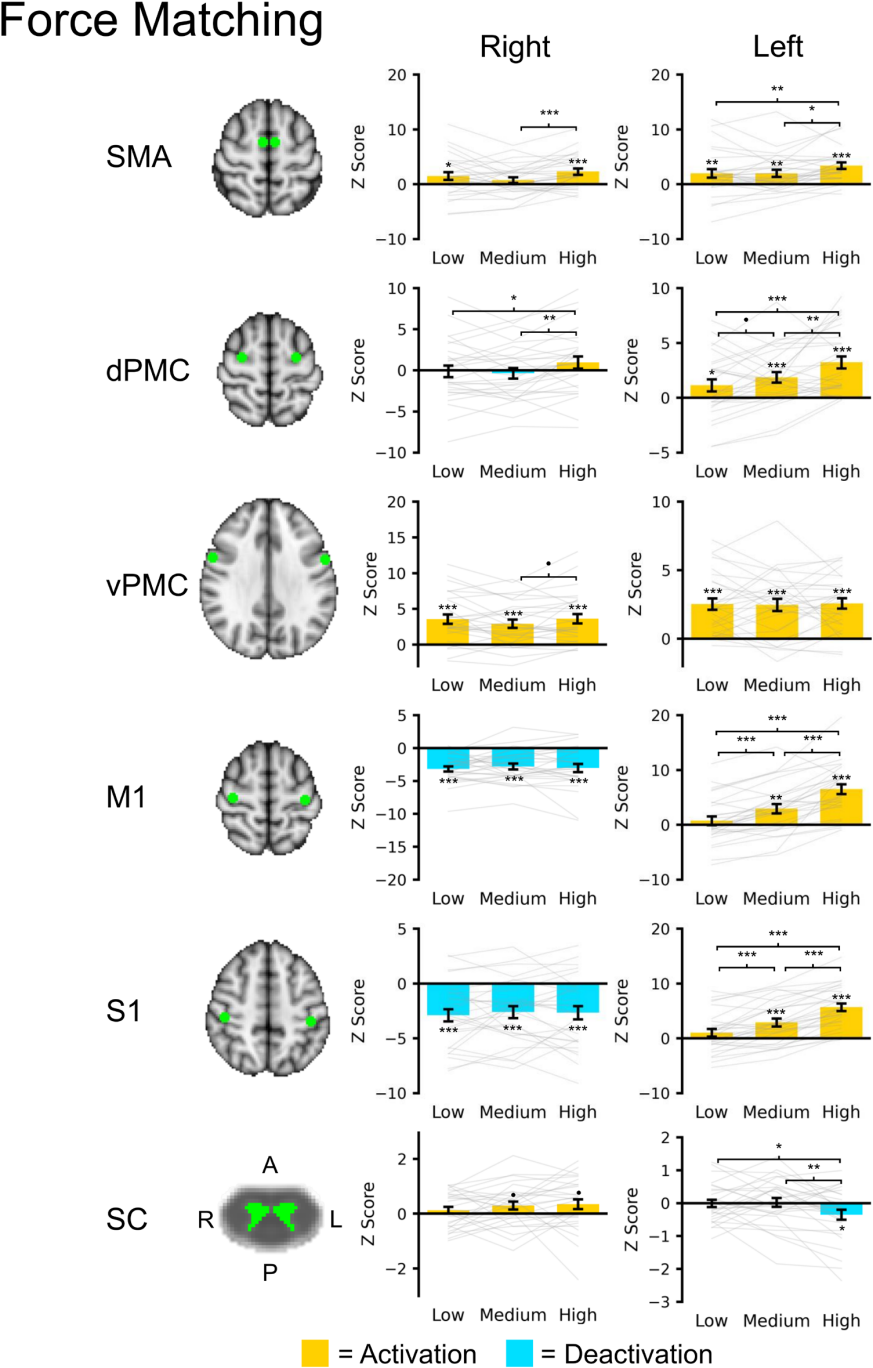
Brain and spinal cord (SC) region of interest (ROI) analysis for the force matching task across the three task levels: low, medium, and high. For the brain ROIs, spheres (radius = 5 mm) were placed at the supplementary motor area (SMA), dorsal premotor cortex (dPMC), ventral premotor cortex (vPMC), primary motor cortex (M1), and primary somatosensory cortex (S1). The spinal cord ROIs included the left or right gray matter spanning the C5 to C6 spinal cord segment levels. The brain and spinal cord regions are shown in green overlaid the respective template. The average Z score within each ROI was extracted. Bar plots show the average activation (yellow) or deactivation (blue) within each region across the subjects. Gray lines show subject level activity level across the task levels. Error bars ± 1 SE. •p < 0.10, *p < 0.05, **p < 0.01, and ***p < 0.001.

ROI analysis revealed bilateral activation of the left and right SMA, present across all task levels with the exception of the right SMA during the medium task level (Fig. 4). SMA activation was highest during the high task level. The left dPMC showed a graded increase in activation across the task levels, while the right dPMC was only activated during the high task level. The left and right vPMC were activated at a largely consistent magnitude across the task levels. The left M1 and S1 were activated during the medium and high task levels with a graded increase in activation across the task levels. Interestingly, the right M1 and S1 showed a consistent magnitude of deactivation across the task levels, consistent with contralateral inhibition. The right spinal cord gray matter showed a trend toward activation for the medium and high task levels, but the magnitude of activation did not reach statistical significance. However, deactivation was seen in the left spinal cord gray matter during the high task level. A positive trend between left M1 activity and right spinal cord GM activity was present but did not reach statistical significance (β = 0.034 ± 0.018, t = 1.954, p = 0.054), and a negative association between left M1 activity and left spinal cord GM activity was observed (β = -0.039 ± 0.015, t = -2.553, p = 0.013) (Supplementary Fig. S10).

### 3.3 Finger tapping task

**Fig. 5.**
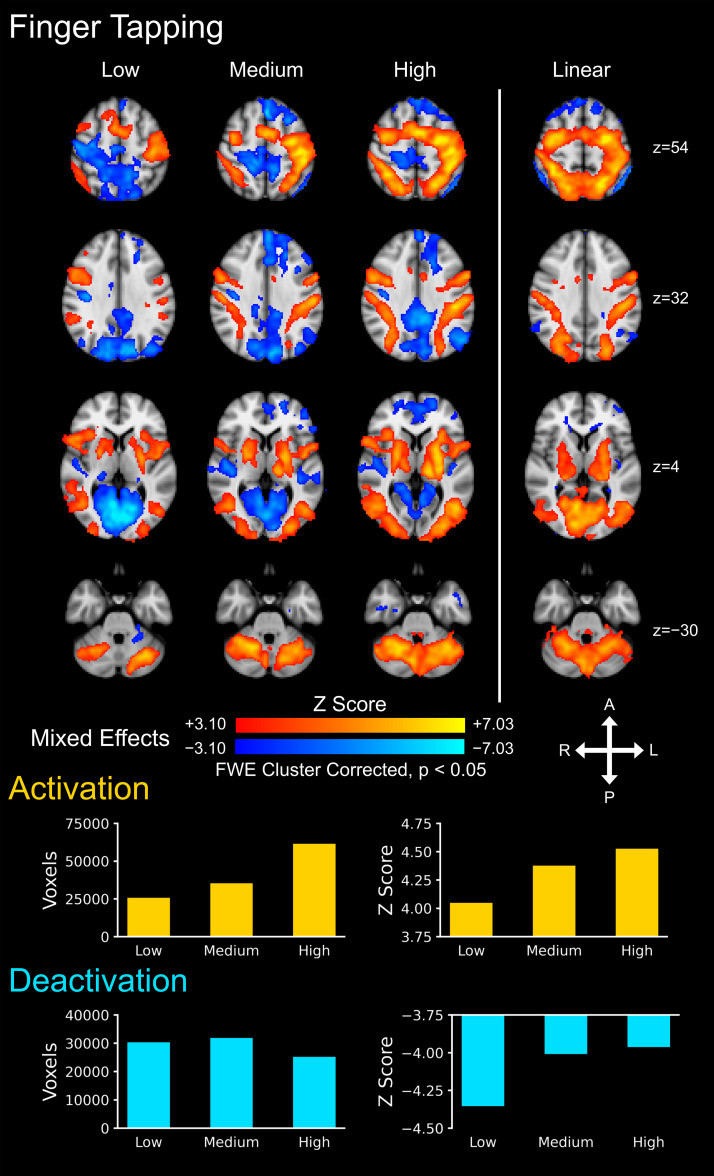
Group level brain activity for the finger tapping task across the three task levels: low, medium, and high. Activations (i.e., positive signal change) are shown in red–yellow and deactivations are shown in blue–light blue (i.e., negative signal change). A linear contrast across the task levels was applied to map where the signal linearly increases and decreases across the task levels. The number of active voxels and the average Z score of the active voxels are shown to summarize the spatial extent and magnitude of the activity across the three task levels. The activation maps were generated from a mixed effects analysis at the group level and were voxel-wise thresholded at a Z score > 3.10 with a family-wise error (FWE) cluster correction threshold of p < 0.05. The background image is the MNI152 T1-weighted brain template. A = anterior, P = posterior, L = left, R = right.

Brain activity maps for the finger tapping task are presented in Figure 5. Activations and deactivations were seen in cortical and subcortical motor and sensory areas at each task level, including the contralateral M1 and S1, bilateral SMA, and bilateral vPMC (mixed effects, Z > 3.10, cluster correction threshold p < 0.05). At the group level, the number of active voxels and the average Z score of the activations increased across the task levels. The number of active voxels for the deactivations was lowest for the high task level, and the average Z score of the deactivations decreased across the task levels.

**Fig. 6.**
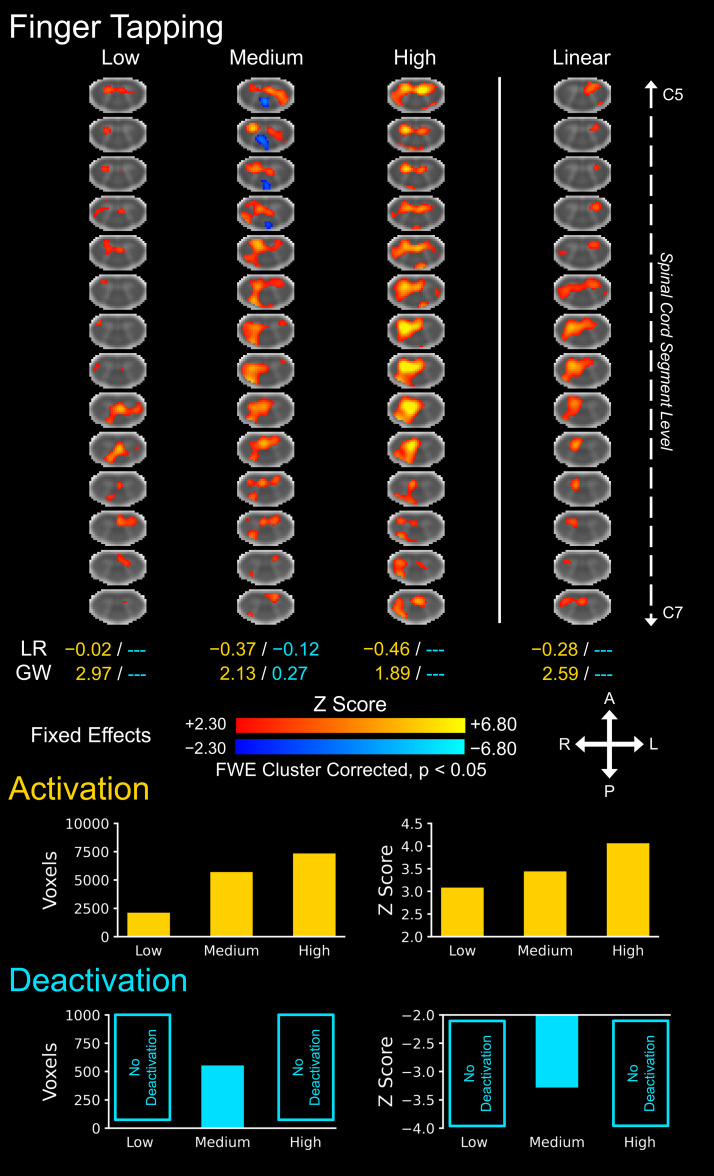
Group level spinal cord activity for the finger tapping task across the three task levels: low, medium, and high. Activations (i.e., positive signal change) are shown in red–yellow and deactivations are shown in blue–light blue (negative signal change). A linear contrast across the task levels was applied to map where the signal linearly increases and decreases across the task levels. The location of the activations and deactivations was assessed using the left–right (LR) index and gray matter–white matter (GW) ratio (--- = no activity, unable to calculate). The number of active voxels and the average Z score of the active voxels are shown to summarize the spatial extent and magnitude of the activations and deactivations across the three task levels. The activation maps were generated from a fixed effects analysis at the group level and were voxel-wise thresholded at a Z score >2.30 with a family-wise error (FWE) cluster correction threshold of p < 0.05. The background image is the PAM50 T2*-weighted spinal cord template. Every fifth axial slice from the intersection of the subject level functional images is shown. A = anterior, P = posterior, L = left, R = right.

Spinal cord activity maps for the finger tapping task are presented in Figure 6. Activation was seen in the right ventral and dorsal horns for each task level (i.e., negative LR indices). Deactivation was present only in the medium task level. Activation in the right ventral and dorsal horns linearly increased with increasing task level (fixed effects, Z > 2.30, cluster correction threshold p < 0.05). The activations were localized more to the gray matter (i.e., positive GW ratios) for all task levels and the linear contrast. At the group level, the number of active voxels and the average Z score of the active voxels increased across the task levels. See the Supplementary Material for spinal cord activity maps using a mixed effects analysis as well as maps with and without cluster correction (Supplementary Figs. S5, S7, and S9).

**Fig. 7.**
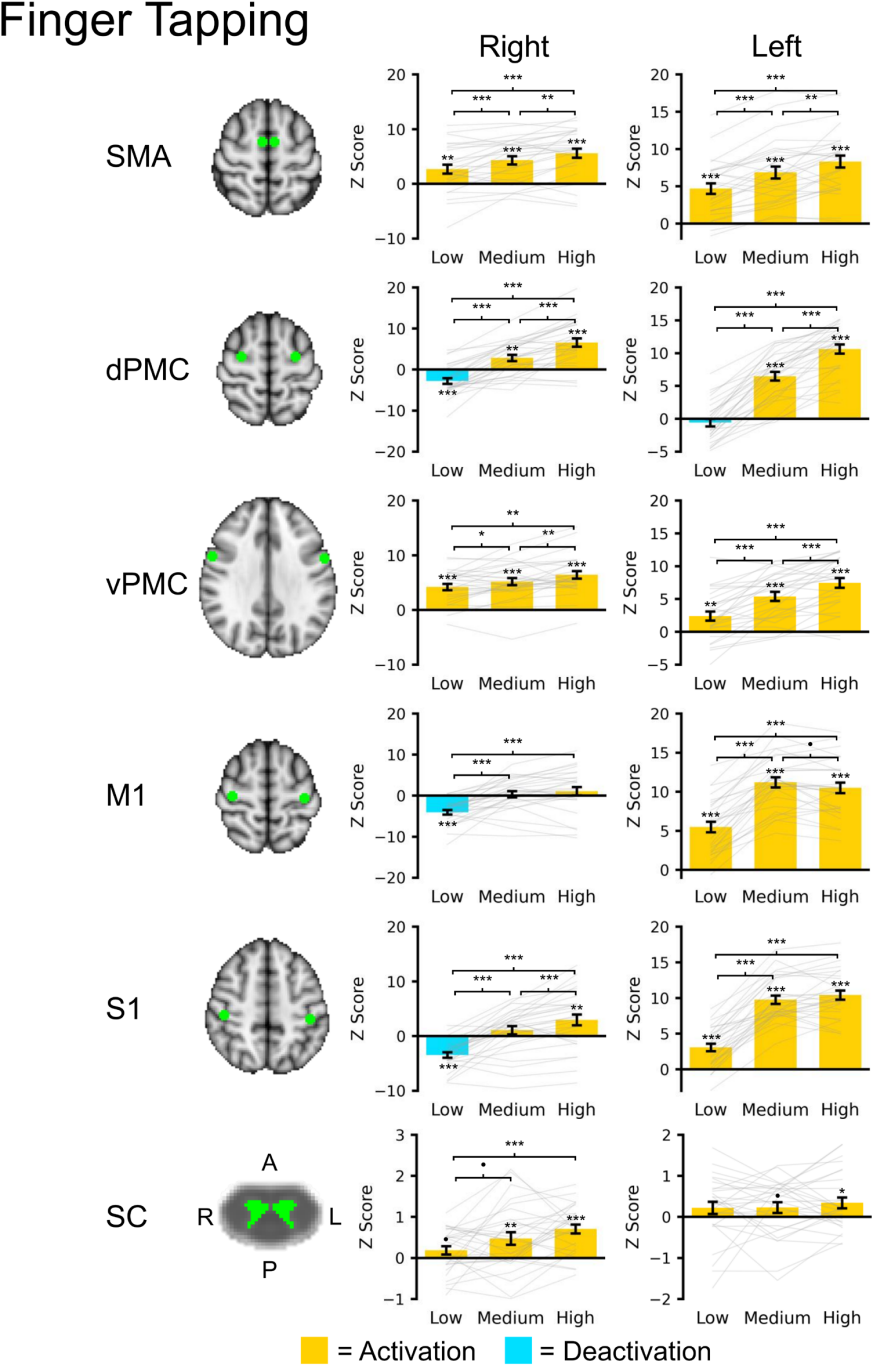
Brain and spinal cord (SC) region of interest (ROI) analysis for the finger tapping task across the three task levels: low, medium, and high. For the brain ROIs, spheres (radius = 5 mm) were placed at the supplementary motor area (SMA), dorsal premotor cortex (dPMC), ventral premotor cortex (vPMC), primary motor cortex (M1), and primary somatosensory cortex (S1). The spinal cord ROIs included the left or right gray matter spanning the C5 to C6 spinal cord segment levels. The brain and spinal cord regions are shown in green overlaid the respective template. The average Z score within each ROI was extracted. Bar plots show the average activation (yellow) or deactivation (blue) within each region across the subjects. Gray lines show subject level activity level across the task levels. Error bars ± 1 SE. •p < 0.10, *p < 0.05, **p < 0.01, and ***p < 0.001.

Results from the ROI analysis are presented in Figure 7. The left and right SMA showed a graded increase in activation across the task levels. The right dPMC deactivated during the low task level while the left and right dPMC showed a graded increase in activation across the medium and high task levels. The left and right vPMC also showed a graded increase in activation across task levels. The left M1 and S1 were activated during each of the task levels with higher activation during the medium and high task levels compared with the low task levels, but no statistical difference in activation between the medium and high task levels. The right M1 and S1 showed deactivation for the low task and no activation for the medium task level. The right S1 but not the right M1 was activated during the high task level. The right spinal cord gray matter showed a trend toward activation for the low task level but the magnitude did not reach statistical significance. For the medium and high task levels, the right spinal cord gray matter was activated with no statistical difference in activation between the medium and high task levels. The left spinal cord gray matter showed a trend toward activation for the medium and high task levels, but the magnitude did not reach statistical significance. A positive association between left M1 activity and right spinal cord GM activity was present (β = 0.036 ± 0.017, t = 2.112, p = 0.038), while no significant association between left M1 activity and left spinal cord GM activity was observed (β = -0.006 ± 0.018, t = -0.345, p = 0.731) (Supplementary Fig. S10).

### 3.4 Trial-wise analysis

No consistent linear changes in task error or activity, suggesting the presence of fatigue or motor learning, were identified across the experiments. For the force matching medium task level, task error linearly increased (p = 0.028), and the average Z score of the active voxels in the spinal cord linearly decreased (p = 0.042). No changes in brain activity were identified in the medium task level, and no changes in task error or activity were identified in the low and high task levels (Supplementary Fig. S2). For the finger tapping task, no changes in task error were present across the task levels. For the finger tapping medium task level, the number of active brain voxels linearly decreased (p = 0.029) and the average Z score of the active spinal cord voxels linearly decreased (p = 0.003). No changes in brain or spinal cord activity were identified in the low and high task levels (Supplementary Fig. S3).

## 4 Discussion

We used simultaneous brain–spinal cord fMRI with force matching and finger tapping tasks across three task levels to map and better understand the neural activity related to hand function. In the force matching task, participants gripped a hand dynamometer with their right hand to match three different force levels, while in the finger tapping task, participants completed single-digit responses with their right hand in three different dexterity levels. Across the tasks, we show activity within motor and sensory brain and spinal cord regions with roles in hand movement, which included graded activations and deactivations across the task levels. The observed spinal deactivations may reflect inhibitory mechanisms essential for fine-tuned motor output. These findings have important mechanistic and clinical implications.

Findings presented here align with previous work demonstrating force–dependent M1 activation (Braaß et al., 2023; Cramer et al., 2002; Dai et al., 2001). The ROI analysis complemented the group level maps showing increasing activation of the left M1 and S1 with increasing force generation. M1 is the main brain region for descending motor commands, which makes monosynaptic connections to motor neurons in the spinal cord ventral horn, via the corticospinal tract, leading to muscle contraction. Motor units (a motor neuron and associated muscle fibers) are recruited in a graded fashion based on size and force production where smaller, lower force generating motor units are recruited first followed by larger, higher force generating motor units (i.e., Henneman’s size principle (Henneman, 1957)). The left M1 activity scaled with force to produce the descending drive needed to recruit the appropriate motor units and achieve the target force levels. Interestingly, we revealed that S1 also scaled with force. In addition to receiving ascending sensory information, S1 projects to the spinal cord to modulate motor activity and sensory processing based on sensory feedback (Karadimas et al., 2020; Ueno et al., 2018). The graded S1 activity represents the increasing tactile and proprioceptive aspects of the tasks as well as descending modulation. For the finger tapping task, the group level brain activity showed graded activation of the left (i.e., contralateral) M1 and S1, which linearly increased across the task levels. The ROI analysis complements the group level maps but shows a clear step in the magnitude of left M1 and S1 activation from the low task level to the medium and high task levels with the magnitude of activation being largely consistent between the medium and high task levels. The increase in M1 and S1 activity between the low task level, second digit only, and medium and high task levels, all digit tapping (sequential or random order), may represent spatial summation within M1 and S1, as all digit tapping requires activation of more motor units to coordinate the movement. Similarly, all digit tapping spatially activates more afferents than single digit tapping, leading to greater S1 activation. A novel interpretation of this finding is that single-digit tapping of the second digit could be driven more by spinal central pattern generators, neural circuits that produce rhythmic motor activity without descending input (Emanuelsen et al., 2018; Steuer & Guertin, 2019; Zehr et al., 2004). If so, less descending drive would be needed for the second digit tapping than all digit tapping, since the rhythmic muscle activation would be spinally mediated, and only descending commands to start and stop the tapping would be needed. The SMA, dPMC, and vPMC are higher level motor areas involved in the planning and organization of motor activity (Wong et al., 2015). These regions receive multisensory information and develop a motor plan that is delivered to M1, which in turn activates the specific muscles. The group level brain activity and ROI analysis showed graded activation of the SMA in both the force matching and finger tapping tasks with more pronounced bilateral activation in the finger tapping task. Graded SMA activation has been reported in similar force matching tasks, highlighting its potential role in force generation (Galléa et al., 2008). The SMA is involved in planning complex movements and sequence processing including the order and timing of movements, explaining the graded activation in the finger tapping task. Likewise, SMA activation was highest for tapping in a random order, which requires the most attention and planning (Cona & Semenza, 2017; Nachev et al., 2008). DPMC directs visually guided behavior and plans movements in response to visual cues while the vPMC is involved in the perception of space, object manipulation, and transforming positions in space to upper limb movements (Bonnard et al., 2007; Davare et al., 2006; Hoshi & Tanji, 2007). During the force matching task, participants were instructed to keep the force bar within ±1 kgf of the target force. Therefore, the target range at the high task level was smaller relative to the target force than at the low task level. The higher task levels required more visual feedback and attention to match the target force level, which could explain the graded left dPMC activity. The dPMC and vPMC showed increasing bilateral activation across the task levels in the finger tapping task, supporting their roles in planning movements to visual cues and spatial perception, respectively. The patterns of SMA, dPMC, and vPMC activity were largely similar bilaterally with the exception of the right dPMC, which was only activated in the high task level of the force matching task. While only a unilateral task was performed here, the bilateral activation may be due to their roles in planning and coordinating complex movements involving both sides of the body.

The right ventral horn of the spinal cord contains the motor neurons that innervate muscles controlling the right hand. For the force matching task, the group level spinal cord activation maps show right ventral and dorsal spinal cord GM activation with evidence of graded activity across the task levels as expected (Braaß et al., 2023; Madi et al., 2001). Results from the ROI analysis, which averaged activity across the C5 to C7 spinal cord segments, are less clear but provide evidence of lateralized right-sided activity with force generation, as expected (Hemmerling et al., 2023; Weber et al., 2016). Averaging across all spinal cord segments could hide activation if the motor activity was not localized to a single spinal cord segment and may explain the weaker correspondence between the group level spinal cord maps and ROI analysis. For the finger tapping task, the group level activation maps and ROI analysis also show graded right ventral and dorsal spinal cord GM activation. In comparison, the spinal cord activation in the finger tapping task appears to be greater than the force matching task, which was not expected and in contrast with previous findings (Maieron et al., 2007). A single button press required <5 kgf, which is less than the low task level target force of the force matching task for any participant, and because motor units are recruited based on their size, we expected greater spinal cord activity for the force matching task. Greater activation may be from the dynamic nature of the task, leading to less adaptation and greater spatial summation of motor and sensory activity. Greater activation could be due to the complexity of the descending commands for increasing task level, leading to a greater spinal cord activation. Left M1 activity was significantly positively associated with the right spinal cord GM activity for the finger tapping task, a finding consistent with the known monosynaptic connections between contralateral M1 and ipsilateral spinal cord motor neurons (Bortoff & Strick, 1993; Morecraft et al., 2023; Natali et al., 2024). Based on the spatial analysis, the activity was more localized to the right spinal cord and the spinal cord GM. As seen in previous studies, left spinal cord activation was also present, which may indicate interneuronal processing for the coordination or inhibition of bilateral movements (Maieron et al., 2007; Ng et al., 2008; Stroman & Ryner, 2001; Vahdat et al., 2015; Xie et al., 2009). Being able to inhibit unwanted movements is necessary for normal function (Ferbert et al., 1992; Hanajima et al., 2001), so we also interrogated deactivations within the brain and spinal cord. Consistently, the group level activation maps and ROI analyses demonstrated deactivation of the right M1 and S1, which may be evidence of interhemispheric inhibition to restrict motor output during the unilateral motor tasks. The deactivation of the left spinal cord ventral and dorsal horns (i.e., contralateral motor and sensory spinal cord regions) in the force matching task, seen at the high task level and linear contrast, suggests that the inhibition of motor areas may extend to the spinal cord. In the ROI analysis, the left spinal cord GM was deactivated in the high task level. Further, the magnitude of left M1 activity was negatively associated with the left spinal cord GM activity across the task levels, providing additional indirect evidence of contralateral inhibition. Whether this inhibition results from direct descending modulation from the brain or interneuronal inhibition in the spinal cord remains to be interrogated. Corticospinal projections are complex and synapse on not only motor neurons but also sensory neurons and interneurons, and these projections can be unilateral and bilateral (Ueno et al., 2018). For the finger tapping task, no contralateral deactivation passed cluster correction, but based on the uncorrected maps (Supplementary Fig. S7), deactivation, when present, was more contralateral. With fMRI, we are limited to an indirect measure of neural activity based on the blood hemodynamics and the blood oxygenation level dependent contrast. While negative signal change has been associated with decreased neural activity (Boorman et al., 2010; Shmuel et al., 2006), the functional MRI signal is a combination of excitatory and inhibitory neural signaling, and the meaning of the deactivation (i.e., negative signal change) is unclear (Moon et al., 2021). We are unable to determine whether the increased spinal cord activations or deactivations are due to increased excitatory or inhibitory activity, descending drive, motor neuron activity, sensory feedback, or spinal interneuronal processing. Combining fMRI with electromyography, peripheral nerve stimulation, and transcranial magnetic stimulation could help disentangle the specific neural mechanisms driving the spinal cord signal change. The phenomenon of contralateral spinal cord deactivation (i.e., interhemicord inhibition) requires further replication and interrogation.

Our study has several limitations. Both the extrinsic and intrinsic hand muscles contributed to the force matching and finger tapping tasks, and both tasks also likely required coactivation of the wrist flexors and extensors to increase wrist stiffness and stabilize the wrist. Therefore, the spinal cord activation is expected to span the C6 to T1 spinal cord segments (Dalley & Agur, 2023; Hemmerling et al., 2023). Notably, the C8 spinal segment is associated with finger flexion and extension, which may not be fully captured within the C5–C7 spinal cord segment coverage. While we may have captured the region of peak motor activity, we were not able to fully interrogate the entire cervical enlargement. We have recently developed a 56-channel research-grade head-neck coil, which can improve imaging in the inferior cervical and upper thoracic spinal cord segments. For the force matching task, previous studies have used higher levels of force. After pilot testing, we chose a lower level of force (≤30% of maximum voluntary contraction) to limit fatigue during the 15-min experiment, and *post hoc* analyses did not demonstrate consistent evidence of fatigue. The low level of spinal cord activation seen in the low force matching task level, especially in the ROI analysis, suggests that the activity in the low task level was just above the noise threshold, and starting at slightly higher force level could have improved the detection of low level force activity. Here we modeled the force matching and finger tapping tasks using the ideal task design. Using the measured force, as performed by Hemmerling et al. (2023), or the timing of the button presses as explanatory variables in the first-level analyses could improve the accuracy of the modeling and the detection of brain and spinal cord activity. Additionally, future studies could use electromyography to capture the actual timing and magnitude of the muscle activity, which could be used to characterize the contributions of different muscle groups to the force matching and finger tapping tasks (Khatibi et al., 2022). Bilateral electromyography could also be used to identify or rule out the presence of mirror movements, involuntary movements in the contralateral limb during a unilateral task. Further, we modeled the force matching and finger tapping 15 s trials as a single block. This analysis assumes that the brain and spinal cord activity is constant across the 15 s trial. Future studies may divide each trial into two or more phases to study changes in activity within the trials. Next, we used the PAM50 template in the Spinal Cord Toolbox, and intervertebral disk levels for spatial normalization, which is not ideal as the location of the spinal cord segments (i.e., where the spinal rootlets enter or exit the spinal cord) in relation to the vertebral bodies varies across participants (Cadotte et al., 2015). Matching the participants on the spinal cord neuroanatomy instead of spine anatomy would likely lead to more robust group level analyses. Only the high task level for the force matching and finger tapping task had any activation or deactivation that passed cluster correction with a mixed effects analysis, and the use of a fixed effects analysis for the spinal cord activity maps is a limitation and reduces the generalizability of the group level activation map findings (Supplementary Figs. S4 and S5). We are currently developing techniques for automated identification of the spinal rootlets and spinal cord segment-based spatial normalization methods (Valošek et al., 2024), which should improve the correspondence in spinal neuroanatomy across participants and group level inferences (Bédard,Valošek, et al., 2025).

We believe our study has several notable strengths. Simultaneous brain–spinal cord fMRI and the inclusion of graded force matching and finger tapping tasks allowed for detailed characterization of motor and sensory activity across the central nervous system under varying demands. The findings showing lateralized brain and spinal cord deactivation during unilateral tasks offer novel mechanistic insights into hand motor control and its modulation. Deactivation of the left spinal cord gray matter during the force matching task provides the first evidence that the inhibition of motor areas during a unilateral motor task extends to the spinal cord. Whether this inhibition results from direct descending modulation from the brain or interneuronal inhibition in the spinal cord remains to be interrogated.

In summary, our study advances the understanding of brain–spinal cord interactions in motor function by demonstrating graded neural activation and inhibition across the neuraxis. By integrating brain and spinal cord imaging, we provide a comprehensive model of sensorimotor control with direct translational relevance. These findings pave the way for future research on targeted neurorehabilitation approaches aimed at optimizing motor function in individuals with neurological impairments.

## Supplementary Material

Supplementary Material

## Data Availability

The code used to analyze the present dataset is available on Github: https://github.com/NeuromuscularInsightLab/Oliva_Mapping_Hand_Functon_Imaging_Neuroscience_2025. The data analyzed in the present study is freely available on OpenNeuro: https://openneuro.org/datasets/ds006729.
