# Supplementary material for: Mapping hand function with simultaneous brain–spinal cord functional MRI
Source: Imaging Neurosci (Camb). 2025 Oct 3;3:IMAG.a.159. doi: 10.1162/IMAG.a.159 (PMC12501243; doi:10.1162/IMAG.a.159)
Supplement: Supplementary Material [file IMAG.a.159_supp.pdf]

	Right (x, y, z)	Left (x, y, z)
SMA	6, -4, 56	-6, -4, 56
dPMC	28, -8, 58	-26, -8, 58
vPMC	56, 6, 32	-56, 4, 32
M1	36, -20, 58	-36, -22, 58
S1	44, -26, 50	-42, -30, 50

Table S1 MNI coordinates (x, y, z) of brain regions used in the region of interest analysis.

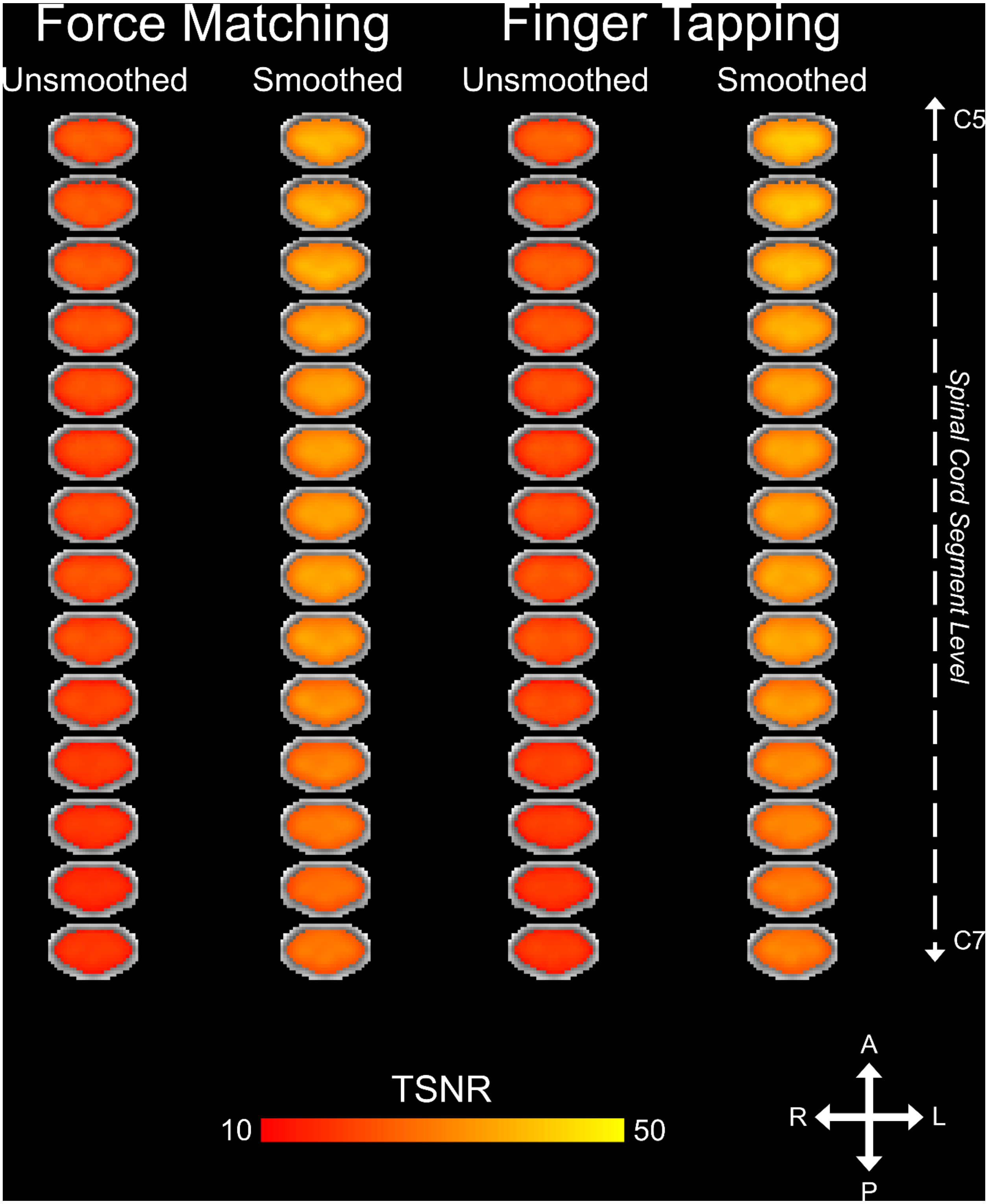

Figure S1. Group level average spinal cord temporal signal-to-noise ratio (TSNR) maps for the force matching and finger tapping tasks with and without spatial smoothing.

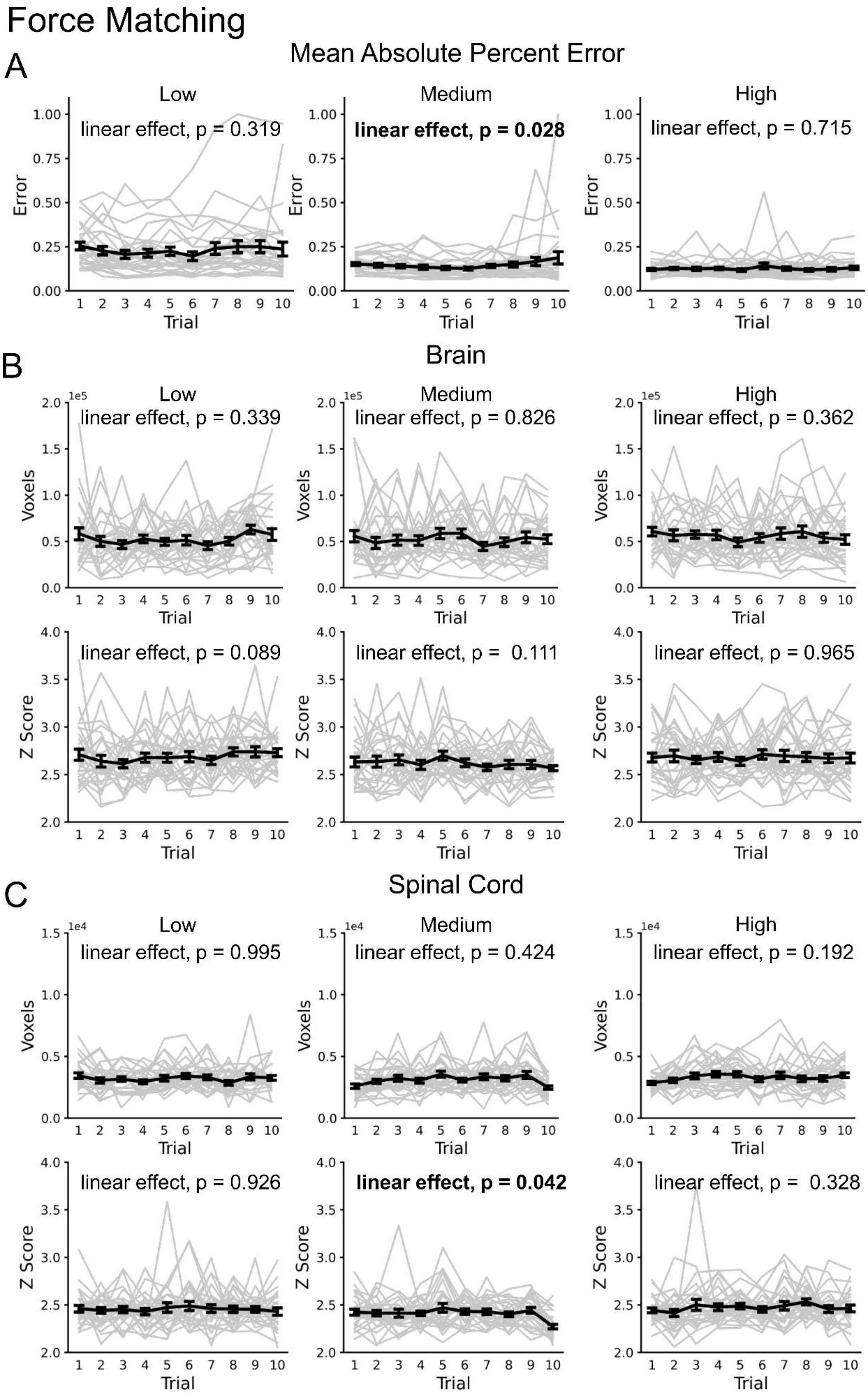
Force Matching
A
Mean Absolute Percent Error
Low
linear effect, p = 0.319
Medium
linear effect, p = 0.028
High
linear effect, p = 0.715
Error
Trial
B
Brain
Low
linear effect, p = 0.339
Medium
linear effect, p = 0.826
High
linear effect, p = 0.362
Voxels
1e5
Trial
linear effect, p = 0.089
linear effect, p = 0.111
linear effect, p = 0.965
Z Score
Trial
C
Spinal Cord
Low
linear effect, p = 0.995
Medium
linear effect, p = 0.424
High
linear effect, p = 0.192
Voxels
1e4
Trial
linear effect, p = 0.926
linear effect, p = 0.042
linear effect, p = 0.328
Z Score
Trial

Figure S2. Trialwise error and brain and spinal cord activation plots (number of active voxels and average Z score of the active voxels) from the force matching task for each trial and task level: low, medium, and high. The average values (± one standard error) across the participants are shown in black. While slight linear increases in the mean absolute error for the medium task level and the number of active spinal cord voxels for the high task level were observed, no consistent linear increases or decreases were present across the experiment, indicating that task performance and activity were largely stationary over the course of the force matching task and no strong evidence of fatigue and motor learning was observed.

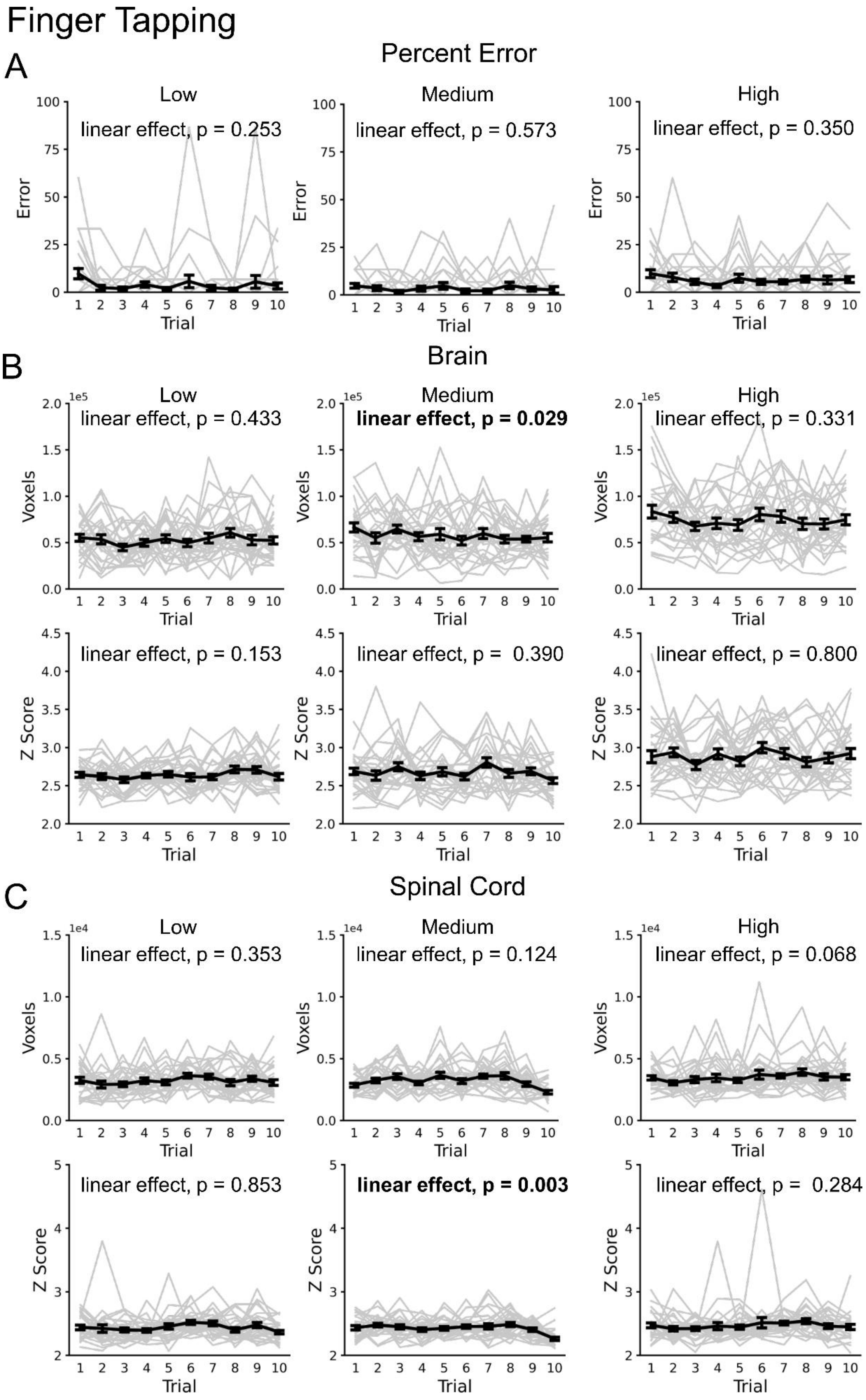
Finger Tapping
A
Percent Error
Low
linear effect, p = 0.253
Medium
linear effect, p = 0.573
High
linear effect, p = 0.350
Error
Trial
B
Brain
Low
linear effect, p = 0.433
Medium
linear effect, p = 0.029
High
linear effect, p = 0.331
Voxels
1e5
linear effect, p = 0.153
linear effect, p = 0.390
linear effect, p = 0.800
Z Score
Trial
C
Spinal Cord
Low
linear effect, p = 0.353
Medium
linear effect, p = 0.124
High
linear effect, p = 0.068
Voxels
1e4
linear effect, p = 0.853
linear effect, p = 0.003
linear effect, p = 0.284
Z Score
Trial

Figure S3. Trialwise error and brain and spinal cord activation plots (number of active voxels and average Z score of the active voxels) from the finger tapping task for each trial and task level: low, medium, and high. The average values (± one standard error) across the participants are shown in black. While slight linear decreases in the number of active brain voxels and the average Z score of the active spinal cord voxels in the medium task level were observed, no consistent linear increases or decreases were present across the experiment, indicating that task performance and activity were largely stationary over the course of the force matching task and no strong evidence of fatigue and motor learning was observed.

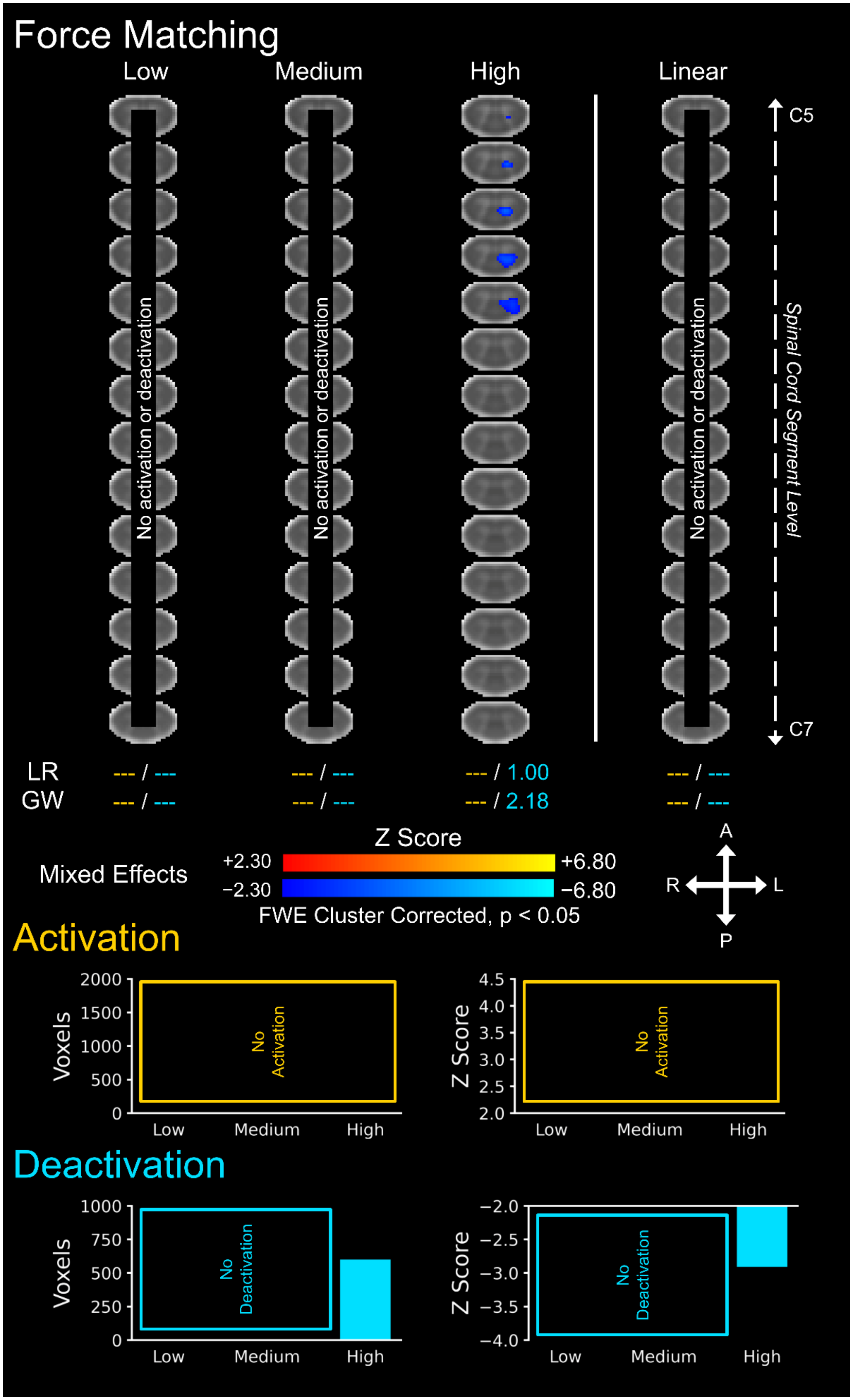

Force Matching
Low
Medium
High
Linear
C5
C7
Spinal Cord Segment Level
No activation or deactivation
No activation or deactivation
No activation or deactivation
LR
GW
--- / ---
--- / ---
--- / 1.00
--- / ---
--- / ---
--- / ---
--- / 2.18
--- / ---
Z Score
Mixed Effects
+2.30
+6.80
−2.30
−6.80
FWE Cluster Corrected, p < 0.05
A
R
L
P
Activation
Voxels
Z Score
No Activation
Low
Medium
High
Deactivation
No Deactivation

Figure S4. Group level spinal cord activity for the force matching task across the three task levels: low, medium, and high. Activations (i.e., positive signal change) are shown in red–yellow and deactivations are shown in blue–light blue (negative signal change). A linear contrast across the task levels was applied to map where the signal linearly increases and decreases across the task levels. The location of the activations and deactivations was assessed using the left-right (LR) index and gray matter-white matter (GW) ratio (--- = no activity, unable to calculate). The number of active voxels and the average Z score of the active voxels are shown to summarize the spatial extent and magnitude of the activity across the three task levels. The activation maps were generated from a mixed effects analysis at the group level and were voxel-wise thresholded at a Z score > 2.30 with a family-wise error (FWE) cluster correction threshold of $p < 0.05$. The background image is the PAM50 T2*-weighted spinal cord template. Every 5th axial slice from the intersection of the subject level functional images is shown. A = anterior, P = posterior, L = left, R = right.

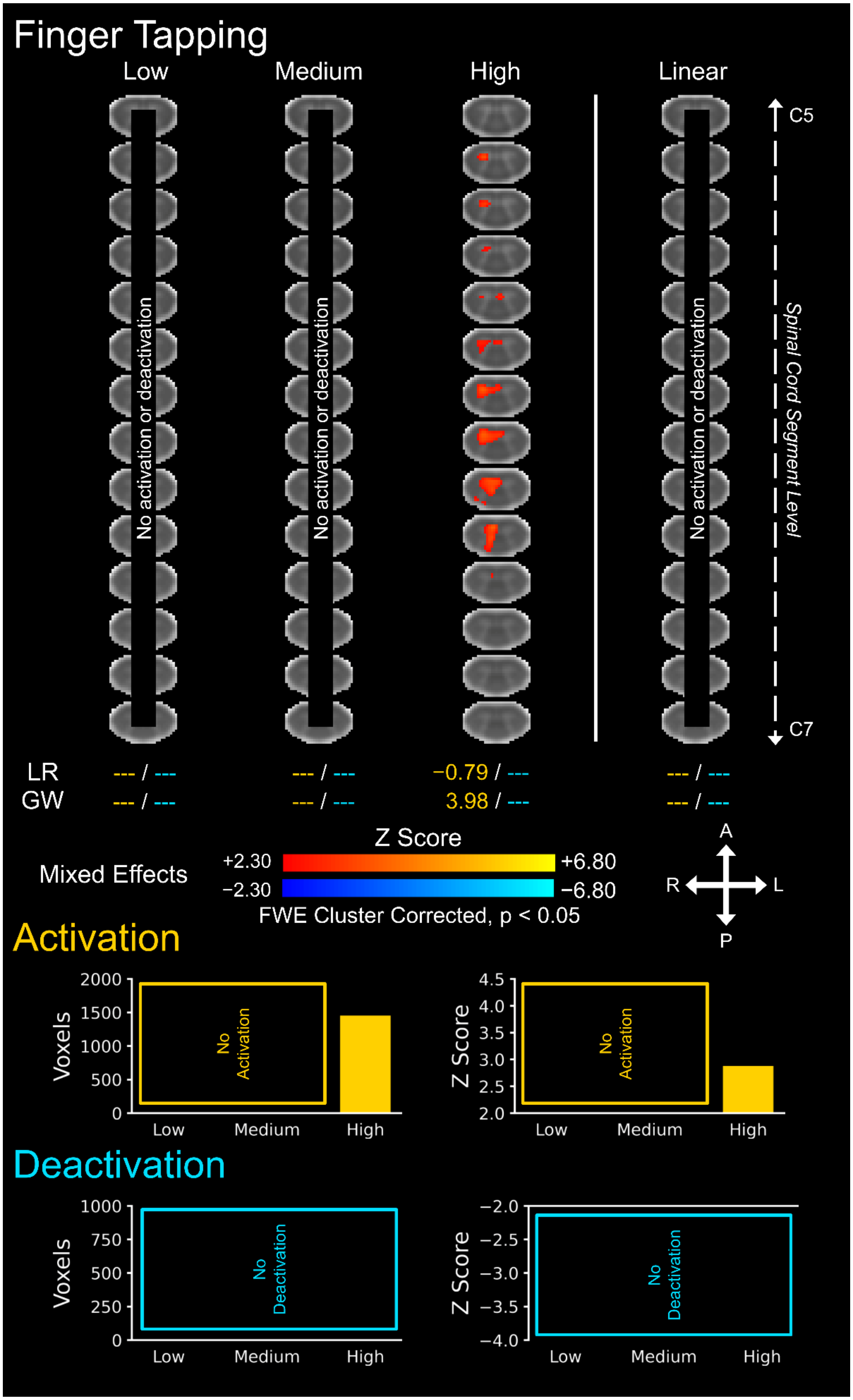

Finger Tapping
Low
Medium
High
Linear
C5
C7
Spinal Cord Segment Level
No activation or deactivation
No activation or deactivation
No activation or deactivation
LR
GW
--- / ---
--- / ---
−0.79 / ---
3.98 / ---
Z Score
Mixed Effects
+2.30
+6.80
−2.30
−6.80
FWE Cluster Corrected, p < 0.05
A
R
L
P
Activation
Voxels
Z Score
No Activation
Deactivation
No Deactivation
Low
Medium
High

Figure S5. Group level spinal cord activity for the finger tapping task across the three task levels: low, medium, and high. Activations (i.e., positive signal change) are shown in red–yellow and deactivations are shown in blue–light blue (negative signal change). A linear contrast across the task levels was applied to map where the signal linearly increases and decreases across the task levels. The location of the activations and deactivations was assessed using the left-right (LR) index and gray matter-white matter (GW) ratio(--- = no activity, unable to calculate). The number of active voxels and the average Z score of the active voxels are shown to summarize the spatial extent and magnitude of the activations and deactivations across the three task levels. The activation maps were generated from a mixed effects analysis at the group level and were voxel-wise thresholded at a Z score > 2.30 with a family-wise error (FWE) cluster correction threshold of $p < 0.05$. The background image is the PAM50 T2*-weighted spinal cord template. Every 5th axial slice from the intersection of the subject level functional images is shown. A = anterior, P = posterior, L = left, R = right. --- = not applicable.

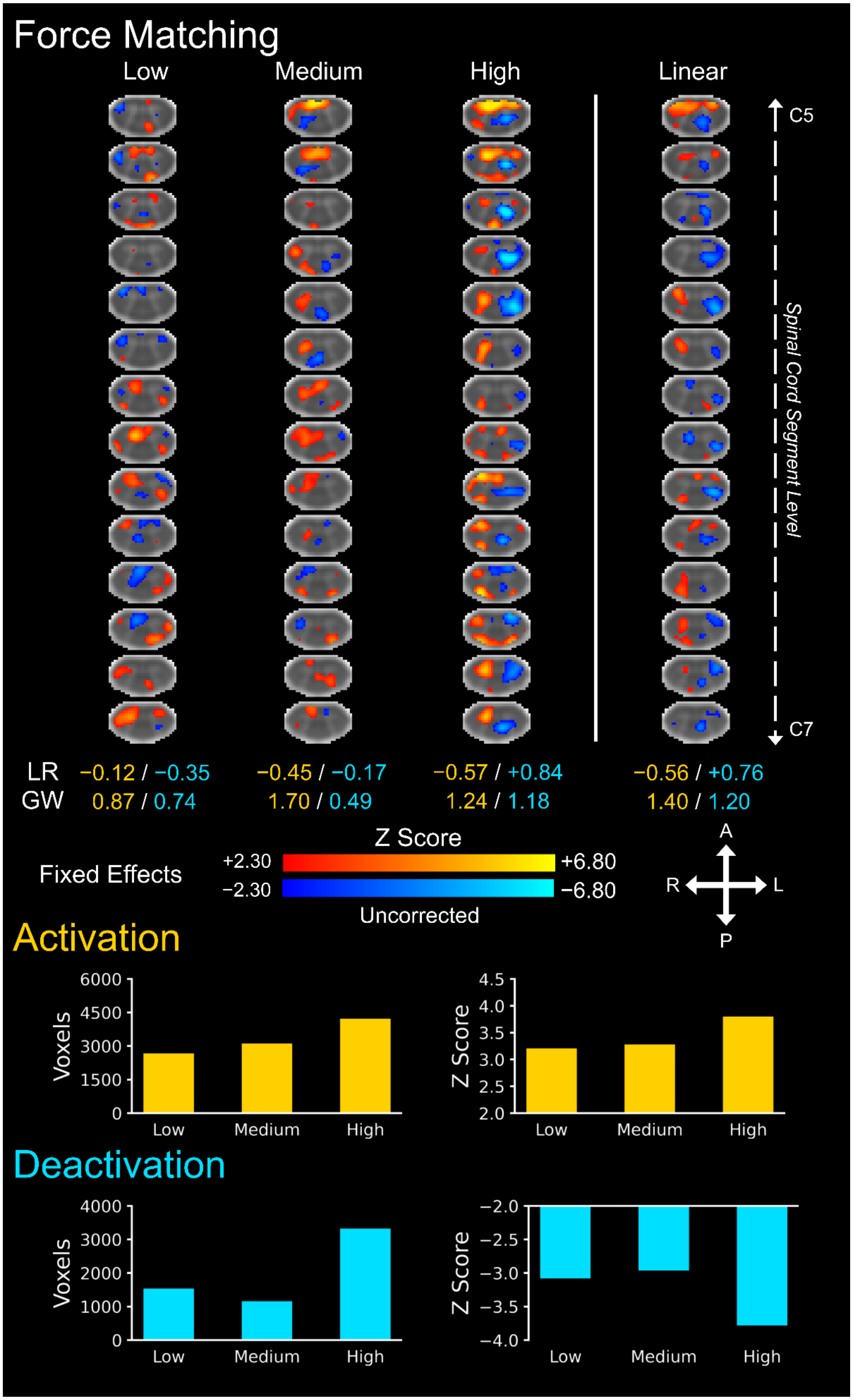

Force Matching
Low
Medium
High
Linear
C5
C7
Spinal Cord Segment Level
LR −0.12 / −0.35
−0.45 / −0.17
−0.57 / +0.84
−0.56 / +0.76
GW 0.87 / 0.74
1.70 / 0.49
1.24 / 1.18
1.40 / 1.20
Z Score
Fixed Effects
+2.30
+6.80
−2.30
−6.80
Uncorrected
A
R
L
P
Activation
Voxels
6000
4500
3000
1500
0
Low
Medium
High
Z Score
4.5
4.0
3.5
3.0
2.5
2.0
Low
Medium
High
Deactivation
Voxels
4000
3000
2000
1000
0
Low
Medium
High
Z Score
−2.0
−2.5
−3.0
−3.5
−4.0
Low
Medium
High

Figure S6. Group level spinal cord activity for the force matching task across the three task levels: low, medium, and high. Activations (i.e., positive signal change) are shown in red–yellow and deactivations are shown in blue–light blue (negative signal change). A linear contrast across the task levels was applied to map where the signal linearly increases and decreases across the task levels. The location of the activations and deactivations was assessed using the left-right (LR) index and gray matter-white matter (GW) ratio(--- = no activity, unable to calculate). The number of active voxels and the average Z score of the active voxels are shown to summarize the spatial extent and magnitude of the activity across the three task levels. The activation maps were generated from a fixed effects analysis at the group level and were voxel-wise thresholded at a Z score > 2.30 without family-wise error correction (uncorrected). The background image is the PAM50 T2*-weighted spinal cord template. Every 5th axial slice from the intersection of the subject level functional images is shown. A = anterior, P = posterior, L = left, R = right.

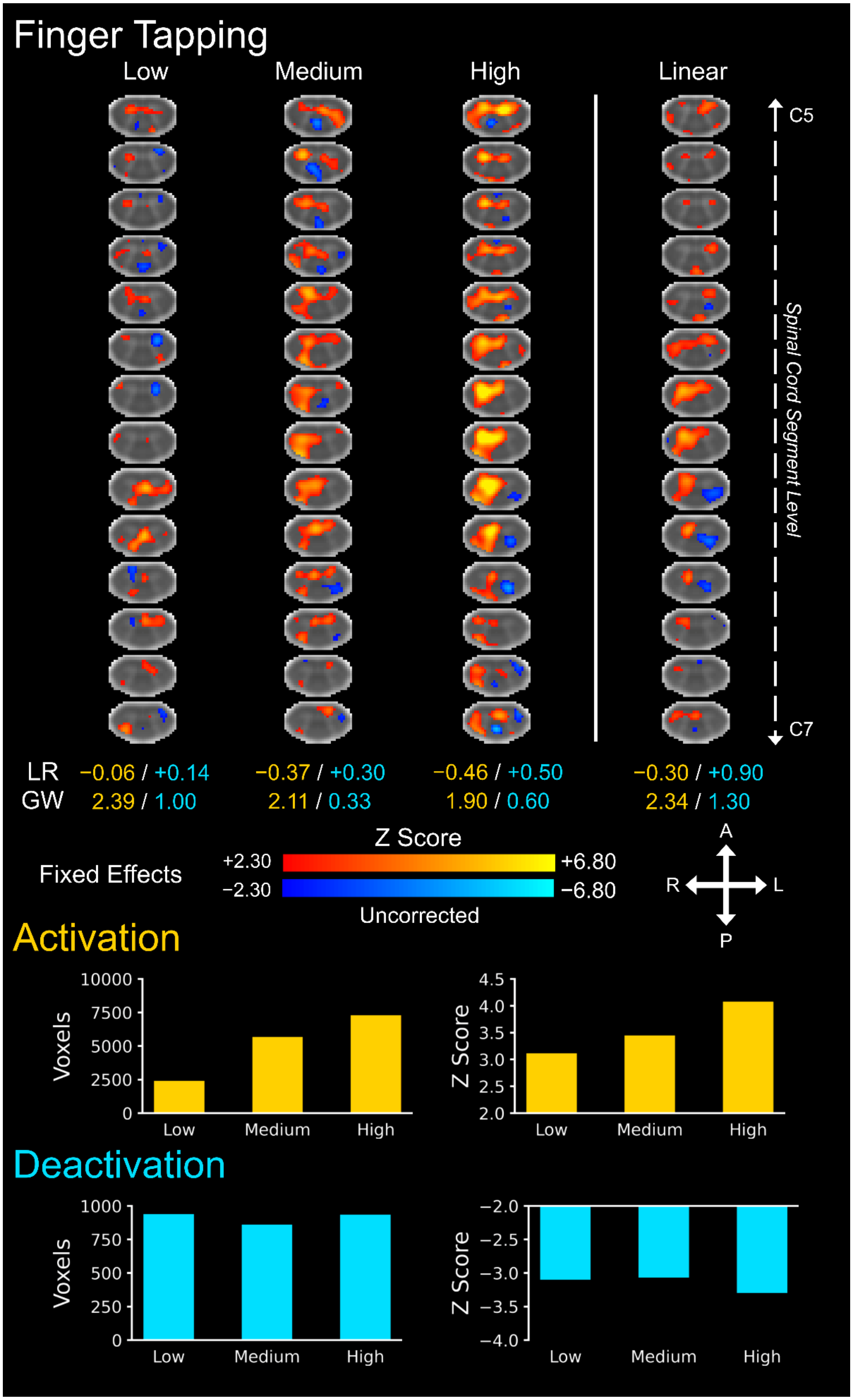
Finger Tapping
Low
Medium
High
Linear
C5
C7
Spinal Cord Segment Level
LR −0.06 / +0.14 −0.37 / +0.30 −0.46 / +0.50 −0.30 / +0.90
GW 2.39 / 1.00 2.11 / 0.33 1.90 / 0.60 2.34 / 1.30
Fixed Effects
Z Score
+2.30
+6.80
−2.30
−6.80
Uncorrected
A
R
L
P
Activation
Voxels
10000
7500
5000
2500
0
Low
Medium
High
Z Score
4.5
4.0
3.5
3.0
2.5
2.0
Deactivation
1000
750
500
250
−2.0
−2.5
−3.0
−3.5
−4.0

Figure S7. Group level spinal cord activity for the finger tapping task across the three task levels: low, medium, and high. Activations (i.e., positive signal change) are shown in red–yellow and deactivations are shown in blue–light blue (negative signal change). A linear contrast across the task levels was applied to map where the signal linearly increases and decreases across the task levels. The location of the activations and deactivations was assessed using the left-right (LR) index and gray matter-white matter (GW) ratio(--- = no activity, unable to calculate). The number of active voxels and the average Z score of the active voxels are shown to summarize the spatial extent and magnitude of the activity across the three task levels. The activation maps were generated from a fixed effects analysis at the group level and were voxel-wise thresholded at a Z score > 2.30 without family-wise error correction (uncorrected). The background image is the PAM50 T2*-weighted spinal cord template. Every 5th axial slice from the intersection of the subject level functional images is shown. A = anterior, P = posterior, L = left, R = right.

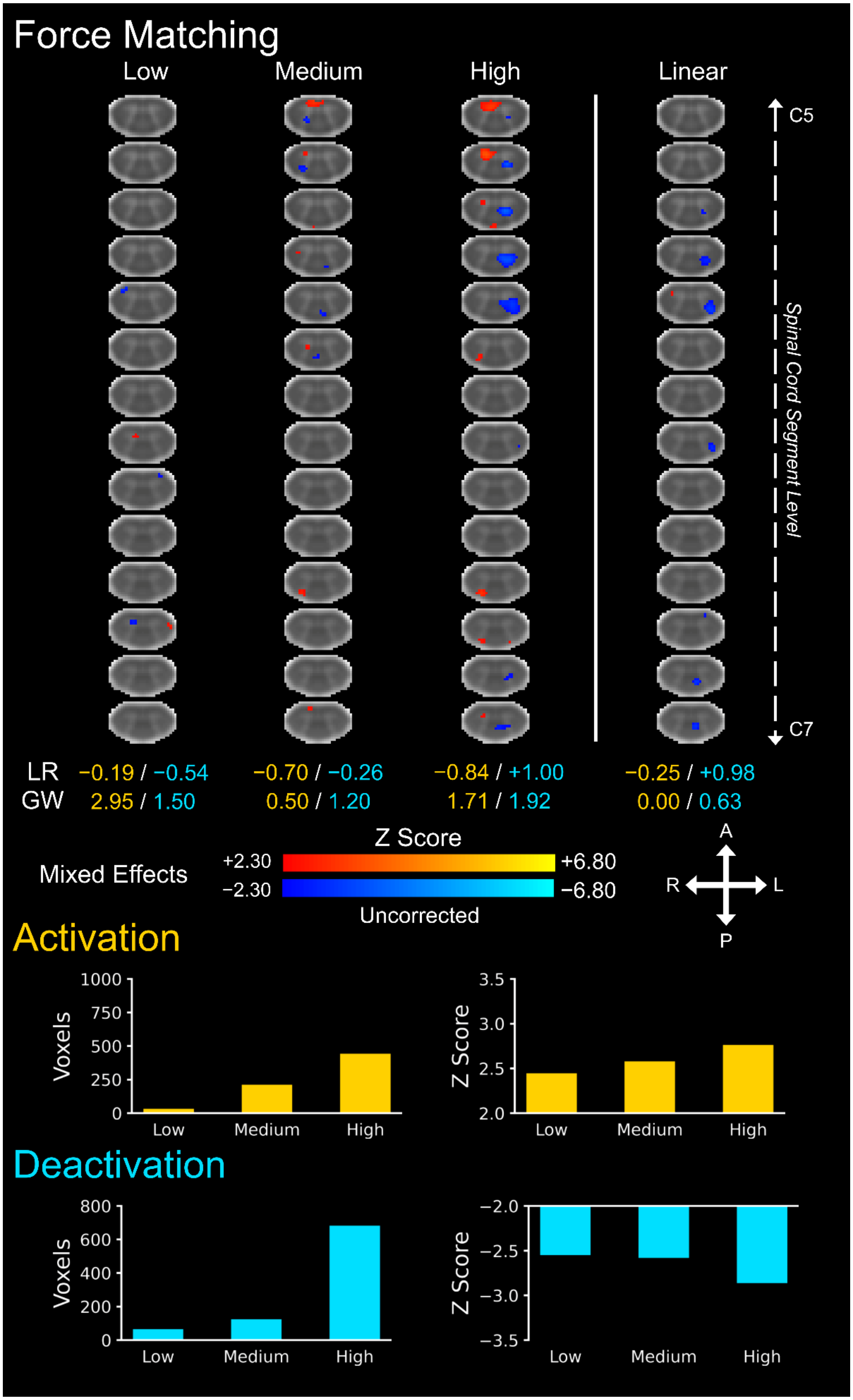

Force Matching
Low
Medium
High
Linear
C5
Spinal Cord Segment Level
C7
LR −0.19 / −0.54 −0.70 / −0.26 −0.84 / +1.00 −0.25 / +0.98
GW 2.95 / 1.50 0.50 / 1.20 1.71 / 1.92 0.00 / 0.63
Z Score
Mixed Effects
+2.30
+6.80
−2.30
−6.80
Uncorrected
A
R
L
P
Activation
Voxels
1000
750
500
250
0
Low Medium High
Z Score
3.5
3.0
2.5
2.0
Low Medium High
Deactivation
Voxels
800
600
400
200
0
Low Medium High
Z Score
−2.0
−2.5
−3.0
−3.5
Low Medium High

Figure S8. Group level spinal cord activity for the force matching task across the three task levels: low, medium, and high. Activations (i.e., positive signal change) are shown in red–yellow and deactivations are shown in blue–light blue (negative signal change). A linear contrast across the task levels was applied to map where the signal linearly increases and decreases across the task levels. The location of the activations and deactivations was assessed using the left-right (LR) index and gray matter-white matter (GW) ratio(--- = no activity, unable to calculate). The number of active voxels and the average Z score of the active voxels are shown to summarize the spatial extent and magnitude of the activity across the three task levels. The activation maps were generated from a mixed effects analysis at the group level and were voxel-wise thresholded at a Z score > 2.30 without family-wise error correction (uncorrected). The background image is the PAM50 T2*-weighted spinal cord template. Every 5th axial slice from the intersection of the subject level functional images is shown. A = anterior, P = posterior, L = left, R = right.

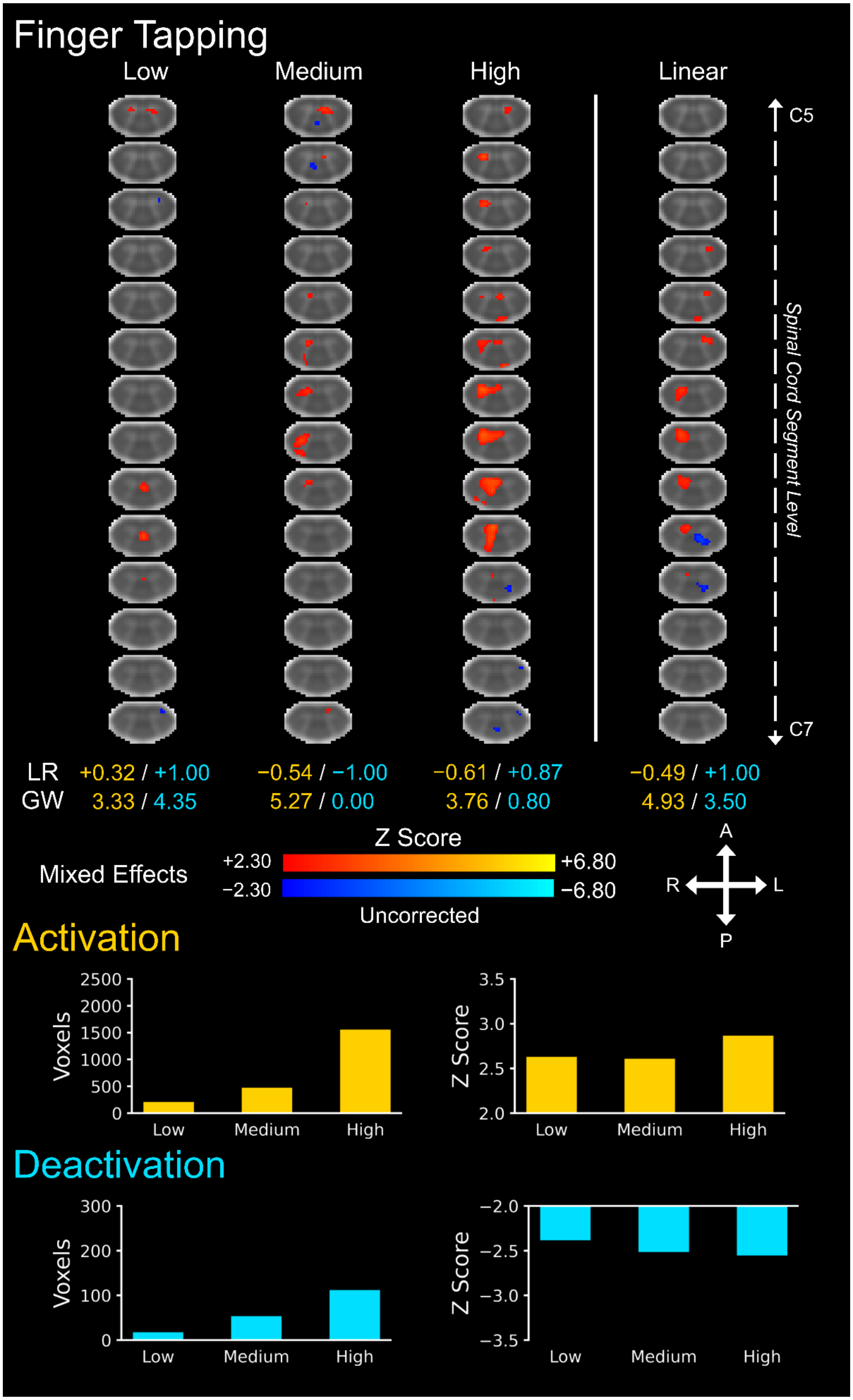
Finger Tapping
Low
Medium
High
Linear
C5
Spinal Cord Segment Level
C7
LR +0.32 / +1.00
−0.54 / −1.00
−0.61 / +0.87
−0.49 / +1.00
GW 3.33 / 4.35
5.27 / 0.00
3.76 / 0.80
4.93 / 3.50
Z Score
Mixed Effects
+2.30
+6.80
−2.30
−6.80
Uncorrected
A
R
L
P
Activation
Voxels
2500
2000
1500
1000
500
0
Low
Medium
High
Z Score
3.5
3.0
2.5
2.0
Low
Medium
High
Deactivation
Voxels
300
200
100
0
Low
Medium
High
Z Score
−2.0
−2.5
−3.0
−3.5
Low
Medium
High

Figure S9. Group level spinal cord activity for the finger tapping task across the three task levels: low, medium, and high. Activations (i.e., positive signal change) are shown in red–yellow and deactivations are shown in blue–light blue (negative signal change). A linear contrast across the task levels was applied to map where the signal linearly increases and decreases across the task levels. The location of the activations and deactivations was assessed using the left-right (LR) index and gray matter-white matter (GW) ratio(--- = no activity, unable to calculate). The number of active voxels and the average Z score of the active voxels are shown to summarize the spatial extent and magnitude of the activity across the three task levels. The activation maps were generated from a mixed effects analysis at the group level and were voxel-wise thresholded at a Z score > 2.30 without family-wise error correction (uncorrected). The background image is the PAM50 T2*-weighted spinal cord template. Every 5th axial slice from the intersection of the subject level functional images is shown. A = anterior, P = posterior, L = left, R = right.

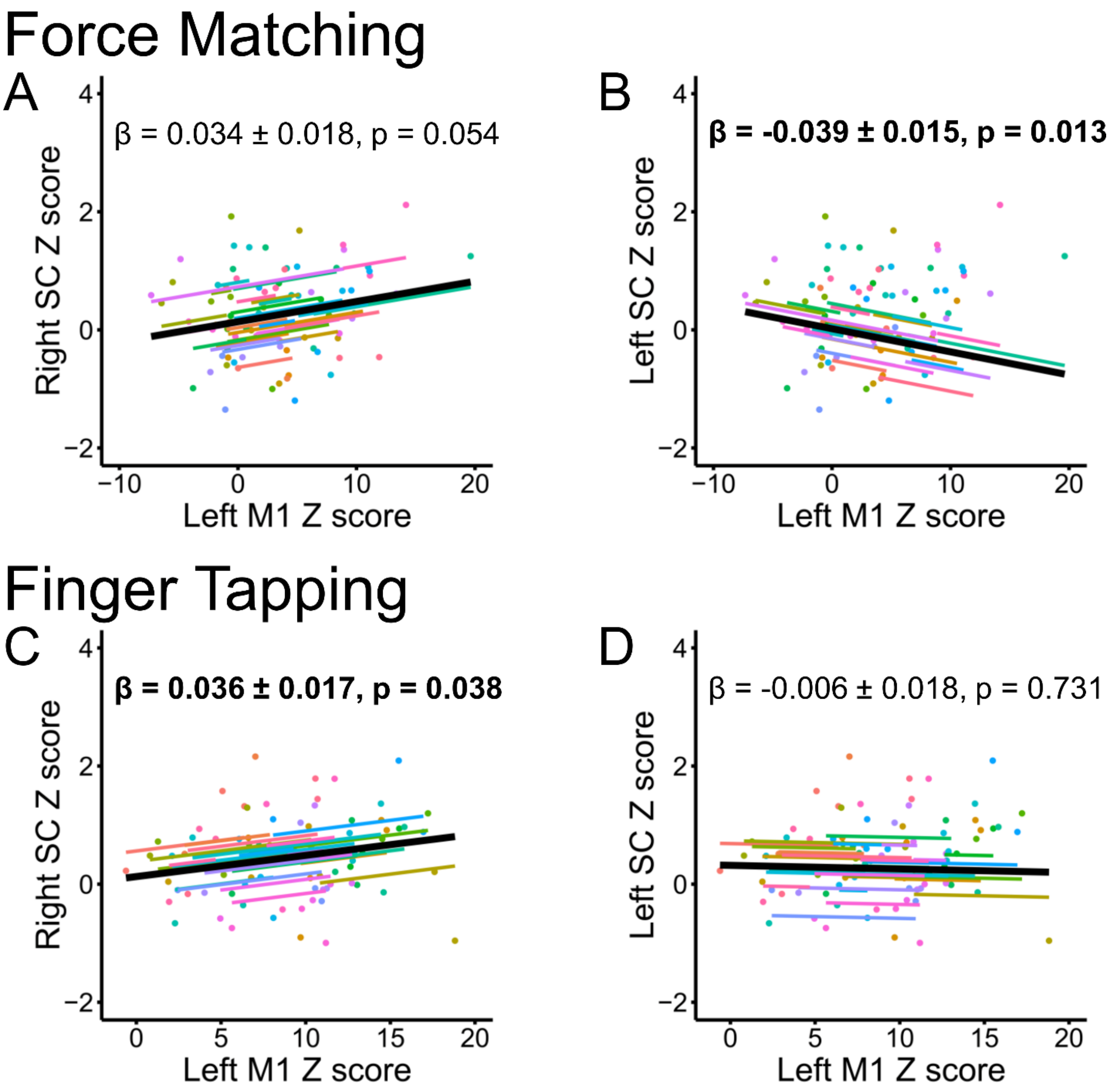

Figure S10. Associations between left M1 activity and spinal cord GM activity across the task levels from repeated measures, mixed effects models.
